# Continuous Topological Transition and Bandgap Tuning in Ethynylene-Linked Acene π-Conjugated Polymers through Mechanical Strain

**DOI:** 10.1021/acs.chemmater.3c02547

**Published:** 2024-01-31

**Authors:** Rameswar Bhattacharjee, Miklos Kertesz

**Affiliations:** Department of Chemistry, Georgetown University, Washington, District of Columbia 20057, United States

## Abstract

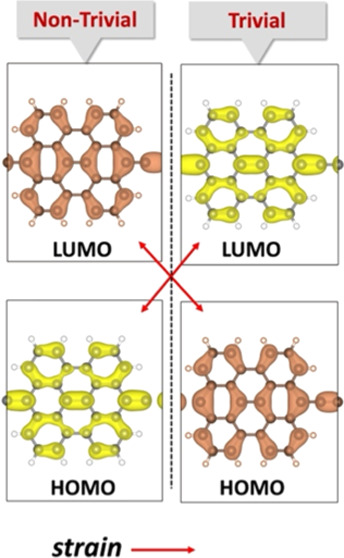

By variation of the chemical repeat units of conjugated polymers, only discrete tuning of essential physical parameters is possible. A unique property of a class of π-conjugated polymers, where polycyclic aromatic hydrocarbons are linked via ethynylene linkers, is their topological aromatic to quinoid phase transition discovered recently by Cirera et al. and González-Herrero et al., which is controllable in discrete steps by chemical variations. We have discovered by means of density functional theory computations that such a phase transition can be achieved by applying continuous variations of longitudinal strain, allowing us to tune the bond length alternation and bandgap. At a specific strain value, the bandgap becomes zero due to an orbital level crossing between the highest occupied molecular orbital (HOMO) and the lowest unoccupied molecular orbital (LUMO). Our hypothesis provides a perspective on the design of organic electronic materials and provides a novel insight into the properties of a continuous phase transition in topological semiconducting polymers.

## 1 Introduction

**Figure 1:**
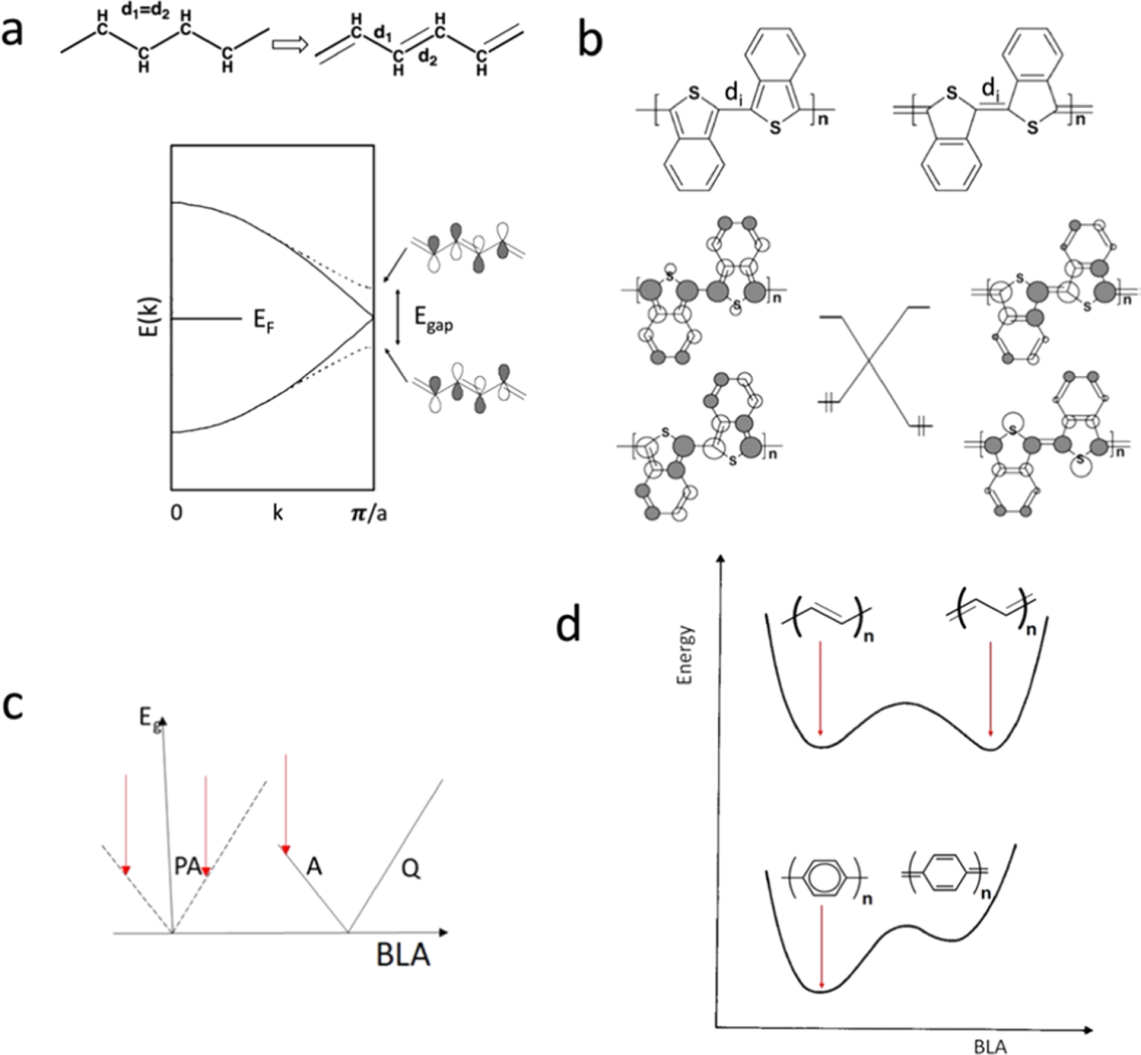
Schematic representation of the bond length alternation (BLA) of polyacetylene and poly p-phenylene, an aromatic ground-state polymer. Arrows indicate the most stable structures with nonzero bandgaps. (a) PA case. (b) Orbital level crossing between an aromatic (A, left) and quinoid (Q, right) structure indicates an opportunity to control the gap by controlling the BLA. (c) Bandgap, E_g_, as a function of BLA for polyacetylene (dashed) and a topological polymer that may have an A or Q ground state (continuous). (d) Symbolic total energy profile for polyacetylene (top) and an A/Q polymer (bottom) as a function of BLA. Red arrows indicate the BLA values of the respective stable structures. Adapted with permission from ref (16). Copyright [2005] [ACS].

The concept of the Peierls distortion,^1^ summarized as “no one-dimensional (1D) metals exist” has been extremely productive in a variety of areas in materials science, including that of conducting conjugated polymers^2^ and other conducting organic materials.^3^ The π conjugation in conjugated polymeric materials provides adjustable bandgaps, making them particularly appealing.^4−12^ A classic example of the Peierls distortion of polyacetylene, PA, leads a transition from a metallic (high-symmetry but nonstable) nuclear configuration to a bond-length-alternating (BLA) lower-symmetry configuration with a sizable bandgap, E_g_, of the order of 1.5 eV,^2^ as illustrated in Figure 1a. (E_g_ is the difference between the energy of the highest occupied molecular orbital (HOMO) and lowest unoccupied molecular orbital (LUMO)). This distortion in PA can be described as a unit cell doubling of a single π-electron per unit cell metal (1/2 filled metallic band) to two π-electrons per unit cell semiconductor. In general, Peierls’s theorem refers to the ubiquitous presence of a vibrational mode in 1D systems that couples to the electronic wave functions near the Fermi level, E_F_, that show the way to an energy-decreasing structural distortion of the lattice preventing a zero-bandgap nuclear configuration to exist. Peierls’s theorem does not specify the size of the lowest achievable bandgap, and numerous efforts in making small bandgap π-conjugated polymers have succeeded in bringing down the bandgap to values as small as 0.9 eV^13^ without violating Peierls’s theorem. In these considerations, BLA has often played a central role. Brédas et al. pointed out, by analogy to the PA case, that in π-conjugated polymers with more complex topology and/or heteroatoms, the bandgap could still be directly controlled by BLA.^14,15^ However, under normal circumstances, Peierls’s theorem intervenes, and a gap of the order of 1 eV results in the most stable structure as illustrated in Figure 1c–d.^16^

**Figure 2:**
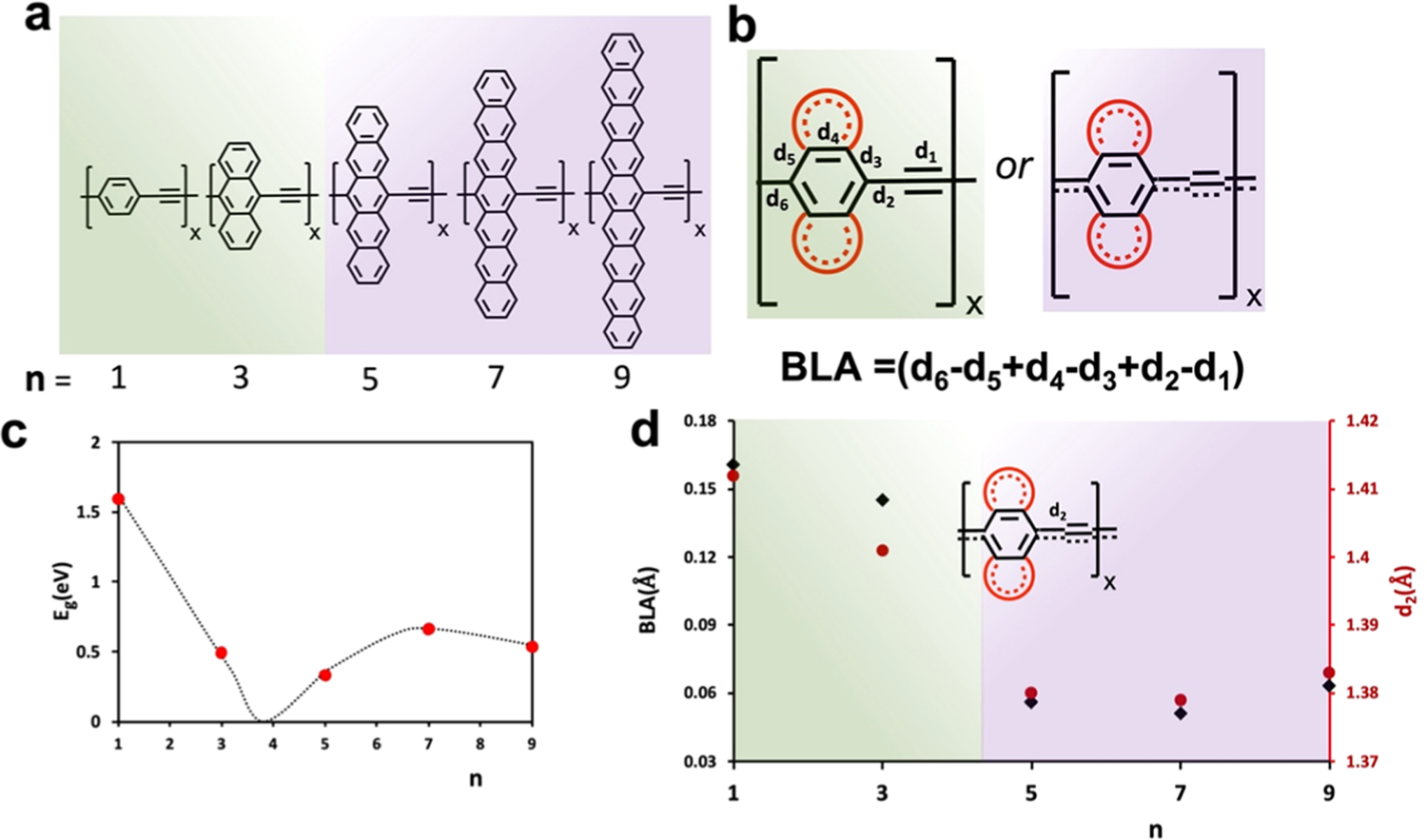
Ethynylene-linked [*n*]acene polymers featuring high π-conjugation. (a) *n* is the number of rings in the acene components. (b) Schematic diagram indicating the BLA formula used here for all members of the series from *n* = 1 to 9. (c) Dependency of the computed bandgap and topological transition between the aromatic (*n* = 1, 3, *Z*_2_ = 0) and quinoid (*n* > 3, *Z*_2_ = 1) ground states of [*n*]acene-yne-linked polymers. (A line is provided to guide the eye.) (d) Computed BLA (black marks) and d_2_ values (red marks) across the series.

An original insight into the behavior of a series of conjugated polymers fabricated on Au(111) surfaces was obtained recently.^17^ In these ethynylene bridged [n]acene polymers, Cirera et al. and González-Herrero et al.^18^ discovered a topological phase transition as a function of the linked acene size (*n*) between anthracene (*n* = 3) and pentacene (*n* = 5) as illustrated in Figure 2.^17^ If *n* were a continuous variable, a small or even zero-bandgap polymer could be contemplated. However, *n* is a *discrete* variable. The polymer closest to the phase transition (*n* = 5) has indeed a very small bandgap of ∼0.35 eV. The topological phase transition in question is between two phases, one with Z_2_ = 0 (trivial, from now on “aromatic” or A) and *Z*_2_ = 1 (nontrivial, “quinoid” or Q) phases,^17^ where *Z*_2_ refers to the topological Zak phase invariant.^19^ In their related paper, they described their discovery of the same phase transition as a function of the oligomer size providing a finer control over the transition.^18^

Can the transition point be approached with an even further reduced gap by introducing a continuously changeable parameter?

**Scheme 1:**
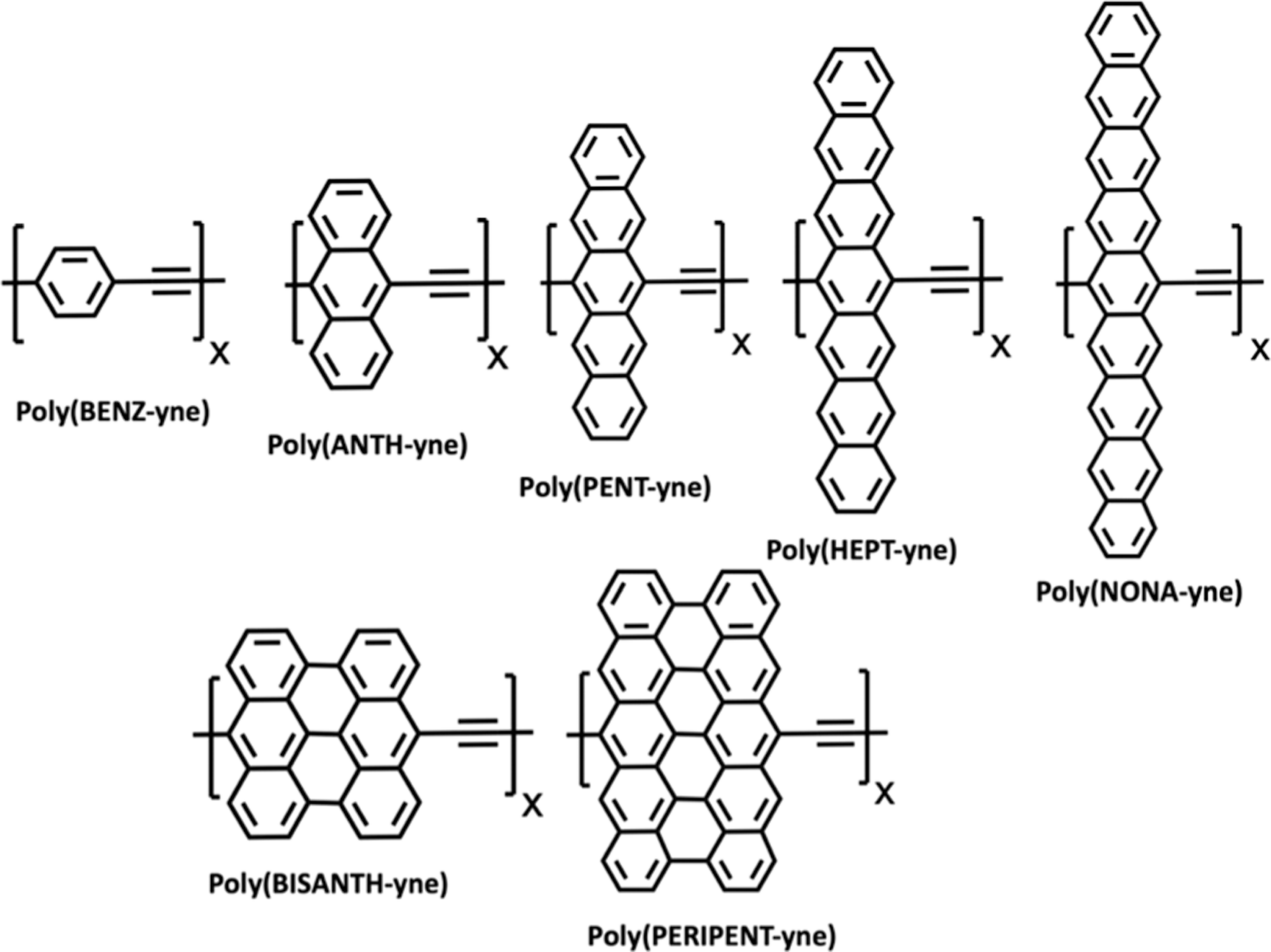
Polymers Discussed in This Work

This review examines a collection of organic-1D polymers that are made up of a sequence of fused aromatic rings linked by an ethynylene unit illustrated in Scheme 1. For the acene-based polymers, we only investigate odd numbers of fused benzene rings, ranging from *n* = 1 to 9 maintaining the D_2h_ symmetry. Additionally, we also consider two periacene repeat units: bisanthene and peripentacene. To simplify the discussion, we identify each polymer with a unique name shown in Scheme 1.

In this paper, we address two aspects of this problem. We have discovered that by introducing the *continuous* variable of mechanical strain, ε, a zero bandgap can be obtained if it is applied to the two systems closest to the topological transition point (*n* = 3 and 5) as determined by Cirera et al.^17^ Then, we address how the external mechanical constraint of the Au(111) surface suppresses the Peierls distortion through the constraint of planarity imposed by the surface-to-polymer interaction. The possibility to tune the bandgap by mechanical strain is connected to the BLA pattern of the presented polymers. As will be discussed below, strain affects different bond lengths differently providing a tool to tune the BLA, and thereby tune E_g_ continuously.

## 2 Results and Discussion

Owing to the extended π-conjugation and unique optoelectronic properties, polycyclic aromatic hydrocarbons (PAHs) have been widely utilized as building blocks in π-conjugated polymers.^20−22^ Bandgap engineering in one-dimensional π-conjugated polymers is a complex and advanced research area, which has resulted in the discovery of new functional organic materials.^23,24^ The discovery by Cirera et al. and González-Herrero et al. comes at the heels of four decades of research into conjugated polymers including significant efforts to engineer their electronic structures through chemical modification some with the goal to reduce the bandgap, E_g_, to the smallest possible value.^15^ In the language of chemistry, many conjugated polymers can exist, at least in principle, in two ground-state structures: aromatic (A) and quinoid (Q), with the latter often characterized by a low bandgap.

The connection between the bandgap of polyacetylene and bond length alternation (BLA) has been established early on^2^ linking this connection with the concept of the Peierls distortion,^1^ which expressed the inevitable energy gain of a bond length alternating geometry compared to a nonalternating (and zero-bandgap) polyacetylene. The connection between a BLA parameter and bandgap is less direct for systems including PAH units or heteroatoms.^16,17^ Nevertheless, since the work by Brédas et al.^15^ on polythiophene and polyisothianaphthene, this connection has been firmly established. They also established that in the case of a large change in the BLA, the structures involved traverse an aromatic vs quinoid structural transition expressing the nature of the interunit cell bond as aromatic, if longer, and quinoid, if shorter. While an unequivocal definition of the BLA parameter is not available if there is more than one π-electron delocalization pathway, in practice, such parameters can be defined also in the presented cases of conjugated polymers, and they correlate well with E_g_. We adopted the definition given in Figure 2b.

### 2.1 Topological Phase Transition and Gap Reduction by Strain

The series of polymers with varying acene size (*n*) groups are shown in Figure 2a, and the generic form is shown in Figure 2b. Figure 2c displays the computed E_g_ values, in agreement with Cirera et al. and González-Herrero et al.^18^ who interpreted the overall V shape behavior as a topological phase transition between two phases, one with the Zak number of *Z*_2_ = 0 (aromatic), and the other with *Z*_2_ = 1 (quinoid).^19^ For the polymer where one benzene ring is linked by the ethynylene linker (poly(BENZ-yne)), the bandgap (E_g_) is the largest at 1.60 eV. When the aromatic unit is replaced by anthracene, the bandgap is significantly reduced to 0.49 eV. This decrease in the bandgap is due to the destabilization of HOMO and some stabilization of LUMO. With increasing size of the aromatic unit to pentacene, heptacene, and nonacene, the trend is reversed due to the HOMO–LUMO level crossing as pointed out by Cirera et al.^17^ This trend is reflected in the BLA values shown in Figure 2d, indicating a switch between two types of BLA values: larger than ∼0.09 Å for aromatic and smaller than that for quinoid structures. Interestingly, the d_2_ bond distances display a very similar correlation, which gives the name for the two types: longer d_2_ for typical aromatic-like molecules, and shorter for the quinoid ones where d_2_ values correspond to typical quinoid-like values.

Since poly(PENT-yne) has the smallest bandgap compared to the other four polymers, we applied longitudinal strain to this system in order to test the hypothesis of whether a continuous topological phase transition can be generated by this perturbation. The length of the optimized equilibrium lattice vector |***a⃗***| for this polymer along the chain direction is 6.933 Å. In the optimized chain, the C≡C triple bond distance is 1.244 Å, which is slightly longer than the equilibrium bond length of 1.203 Å in acetylene. The BLA in poly(PENT-yne) is 0.056 Å at zero strain, ε = 0%.

**Figure 3:**
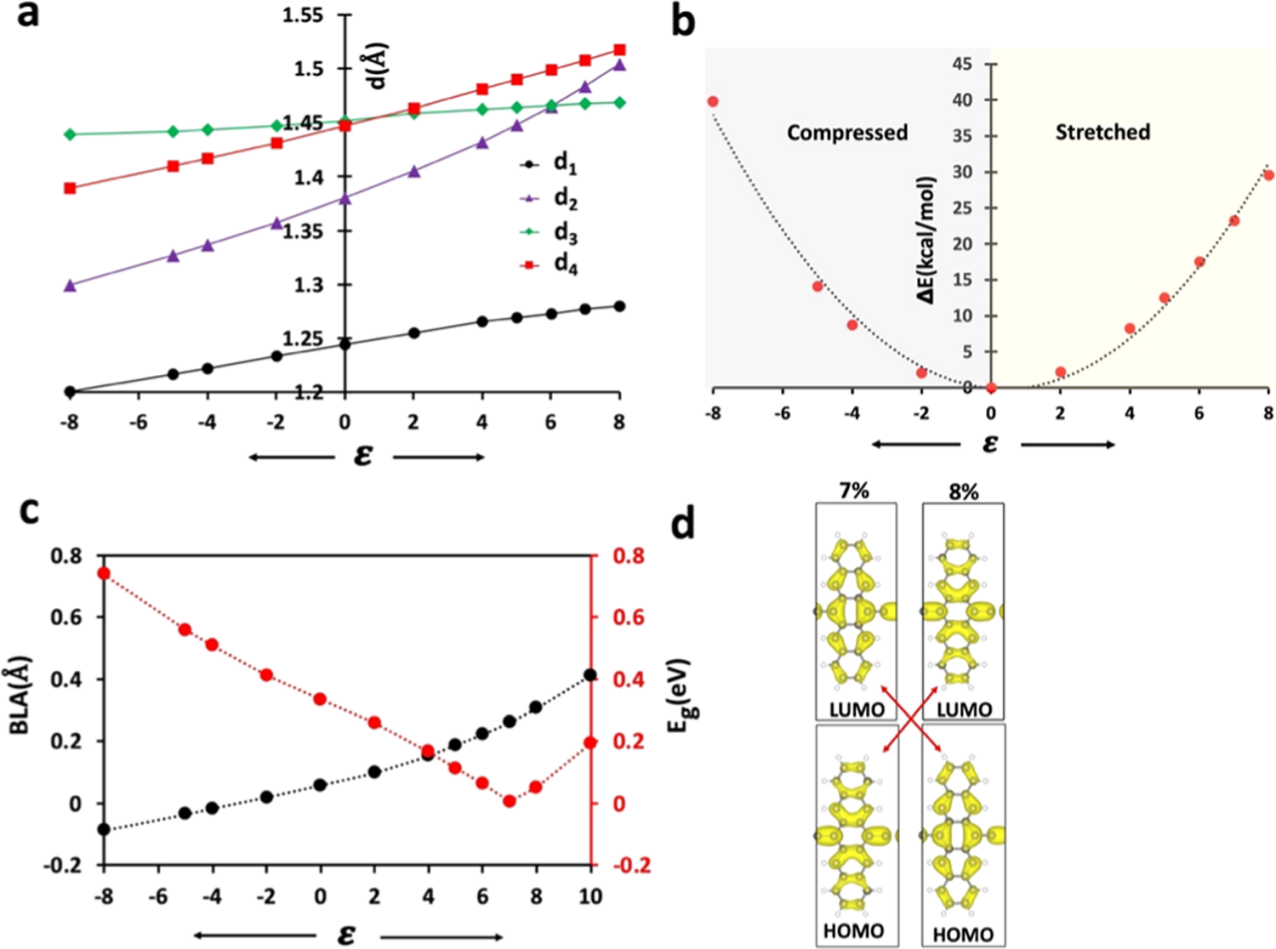
Effect of longitudinal strain, ε, on parameters of poly(PENT-yne). (a) Changes in four relevant bond lengths (d_1_, d_2_, d_3_, d_4_). (b) Relaxed potential energy surface (PES). (c) BLA (in black) and E_g_ (in red). (d) HOMO–LUMO level crossing between ε = 7 and 8%.

Figure 3a illustrates the impact of longitudinal mechanical strain on poly(PENT-yne), highlighting the changes in four key bond distances. The bond length of the linker connecting the ethynylene and the pentacene unit (d_2_) is the most responsive to the strain, elongating from 1.30 to 1.50 Å as the system undergoes a strain change from −8 to +8%. Within the acene part, d_4_ is the most flexible, increasing from 1.39 to 1.52 Å under similar conditions. This can be attributed to its parallel alignment to the applied strain. The change in bond length d_1_ was significantly smaller, ranging from 1.20 to 1.28 Å, due to the large force constant of the C≡C triple bond. Finally, the d_3_ bond length was nearly unaffected by the strain, with a change of only 0.03 Å, as it was directed at an angle of approximately 60° from the axis of the applied strain.

Figure 3b shows the effect of strain on the total energy leading to a realistic Young’s modulus value for single chains, estimated at 113 GPa; see the Supporting Information for details. Most important is the change of E_g_ as a function of strain, which increases upon compression and decreases upon stretching. The bandgap ultimately closes near 7% strain, leading to the metallic behavior of the system (see Figure 3c). Upon further stretching, the bandgap reopens and continues to rise again. The band structures for poly(PENT-yne) at −5, 0, and +7% strain are provided in Figure S3 in the Supporting Information. The strain affected the bandgap of poly(PENT-yne) primarily by perturbing the LUMO, while the HOMO remained nearly unaffected. The presence of a vertical node in the LUMO, illustrated in Figure 3d, serves as a defining factor in this trend. However, stretching the polymer by more than 7% yielded opposite results, where HOMO started to be stabilized to a greater extent. The respective level crossings are illustrated in Figure 3d. Interestingly, the HOMO now has vertical nodes, and further strain will stabilize it further, resulting in a rise of E_g_ (see also Figure S4 in the Supporting Information).

Moreover, we analyzed the effect of strain on poly(PENT-yne) by calculating the BLA values at different strain levels and their relationship with E_g_. The resulting BLA values increased continuously as the polymer was stretched along the chain axis, as shown in Figure 3c with an increasing slope in the positive strain region. E_g_ as a function of BLA exhibits a V-shaped pattern with the level crossing near BLA ≈ 0.26 Å. As has been pointed out, the BLA parameters are generally directly related to E_g_ (see Figure S5a in the Supporting Information), proving that mechanical stress is able to generate the HOMO–LUMO level crossing and thus the topological Q to A phase transition in poly(PENT-yne).

### 2.2 Edge States and the Two Phases

Additional calculations were conducted to characterize the topological phase transition in poly(PENT-yne). These calculations aimed to confirm the Zak-numbers for the trivial (*Z*_2_ = 0) and nontrivial (*Z*_2_ = 1) phases of the system. A critical aspect in discerning the topological phase was identifying midgap states in the form of edge states.^25^ As per the bulk-boundary correspondence (BBC), the presence or absence of these edge states distinguishes the nontrivial from the trivial topological phase.^26^ To identify the edge states, we constructed a long finite chain comprising 15 pentacene units derived from the optimized periodic system at a specific strain. The selection of a 15-unit finite chain aligns with prior research findings.^17^

**Figure 4:**
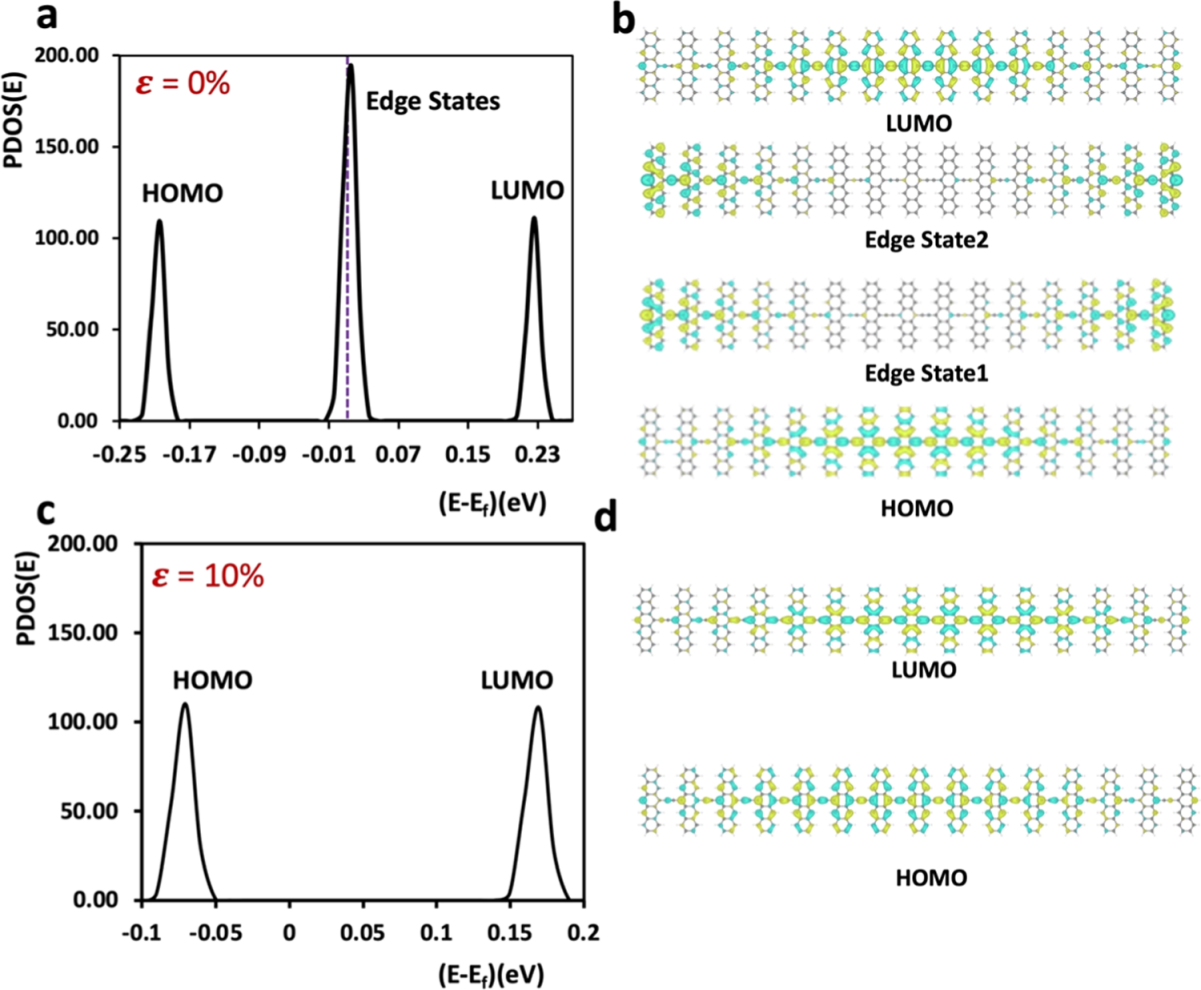
Topological phase transition and topological invariant. (a) Projected density of states (PDOS) for the finite H-terminated poly(PENT-yne) chain comprising 15 units extracted from the optimized periodic structure of poly(PENT-yne) without strain. (b) Frontier molecular orbitals (isovalue= 5 × 10^–5^) of the four bands (edge states are nearly degenerate) depicted in (a). (c) PDOS presentation of a comparable finite H-terminated poly(PENT-yne) chain extracted from the periodic structure of poly(PENT-yne) optimized at 10% tensile strain. (d) Frontier molecular orbitals for the two bands depicted in (c).

At ε = 0%, two nearly degenerate edge states at the Fermi level are present (see Figure 4a), affirming the topological nontriviality of poly(PENT-yne) in its unstressed state in full agreement with the previous observation by Cirera et al.^17^Figure 4b displays the HOMO, LUMO, and two nearly degenerate edge states, in the middle of the HOMO–LUMO gap with wave functions that are localized at the two ends leading to the conclusion that this is a nontrivial (*Z*_2_ = 1) phase. Similar computations were conducted for the poly(PENT-yne) structure on the opposite side of the phase boundary, where the structure is aromatic. Specifically, the structure at ε = 10% was chosen to represent the right side of the topological phase (as depicted in Figure 3c). The edge state bands disappeared (as seen in Figure 4c–d) in the finite poly(PENT-yne), indicating the trivial (*Z*_2_ = 0) phase at that level of stress. This analysis reinforces the understanding that poly(PENT-yne) undergoes a continuous topological phase transition under mechanical strain (ε).

A similar analysis of edge states for poly(BISANTH-yne) follows in the next section, after addressing the effects of strain in that system. Furthermore, the paper later demonstrates that chemical modification induces a topological phase transition in poly(PENT-yne) through a similar analysis.

### 2.3 Further Examples

**Figure 5:**
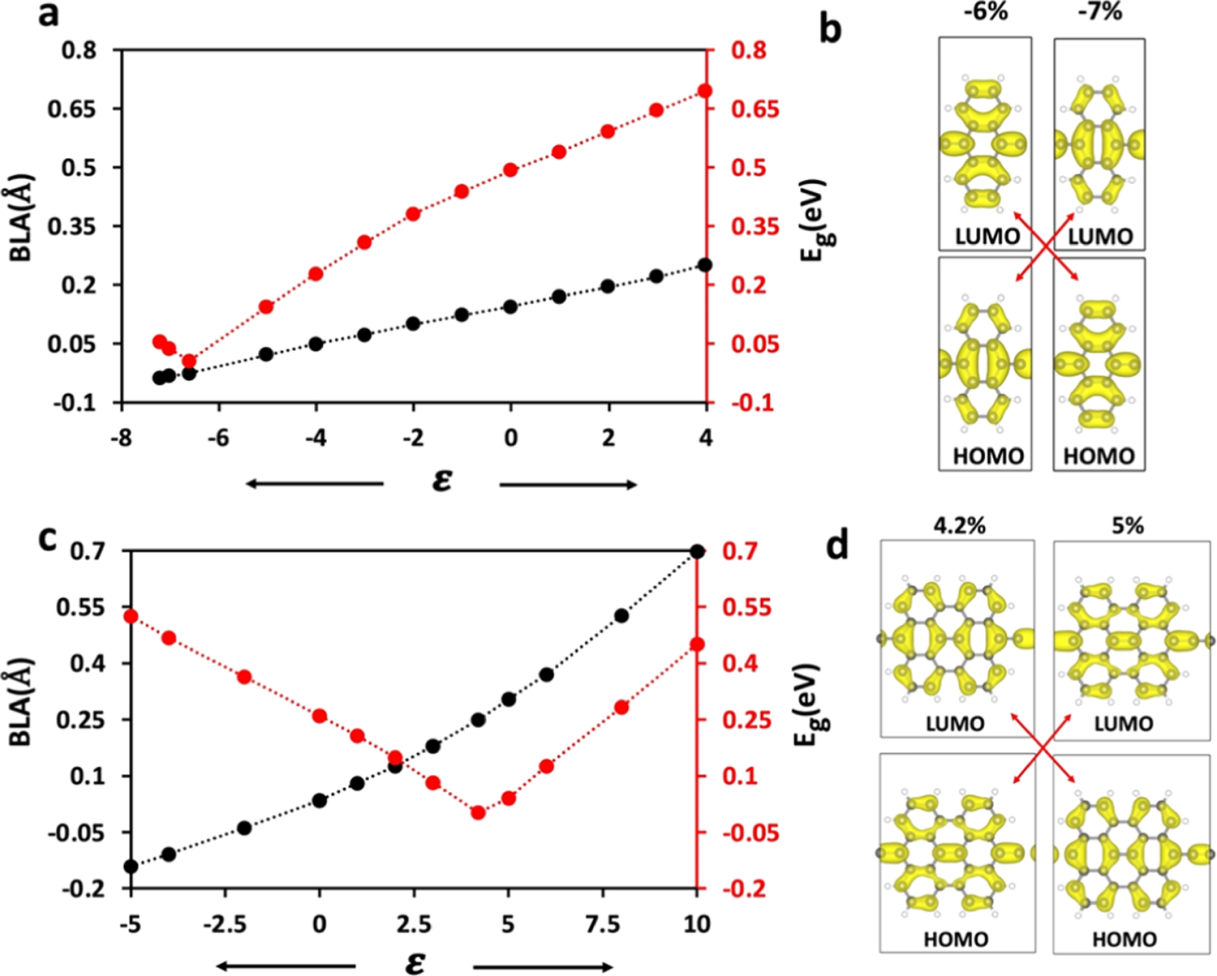
Effect of strain, ε, on parameters of poly(ANTH-yne) and poly(BISANTH-yne) (a) E_g_ (in red) and BLA (in black) of poly(ANTH-yne). (b) HOMO and LUMO of poly(ANTH-yne) before and after the level crossing (c) E_g_ (in red) and BLA (in black) of poly(BISANTH-yne). (d) HOMO and LUMO orbitals of poly(BISANTH-yne) before and after the level crossing. BLA is defined according to Figures 2b and S11 (in the Supporting Information), respectively.

Below we document two more cases of such topological phase transitions as a function of strain, while we point out the reasons for the lack of such transitions in other cases. Poly(BENZ-yne) (*n* = 1) has a much larger E_g_ than the others, so no further calculations were performed on it assuming that the strain required to generate the phase transition would be extreme. The second polymer in Figure 2a is poly(ANTH-yne), which has a bandgap of 0.49 eV at ε = 0%, making it a promising candidate for further attempts to tune its gap by strain. Interestingly, the behavior of poly(ANTH-yne) (*n* = 3) is the opposite to that of poly(PENT-yne) as a function of ε. The bandgap of poly(ANTH-yne) increases when stretched and decreases when compressed, representing a case of aromatic to quinoid topological phase transition. Poly(ANTH-yne) has vertical nodes passing through its HOMO, while LUMO does not have such a type of node, which stabilizes HOMO significantly when poly(ANTH-yne) is stretched, keeping the LUMO almost unchanged, in contrast to the poly(PENT-yne) case. Figure 5a shows the changes in BLA and E_g_ of poly(ANTH-yne) at various levels of strain. The level crossing occurs near ε = −6.6% where the bandgap is completely eliminated, i.e., E_g_ = 0. Further compressing reopens the gap, as expected due to the switch of the frontier orbitals. The relationship between BLA, E_g_, and applied strain exhibits a similar connection to that observed in poly(PENT-yne) (see also Figure S5b in the Supporting Information). Changes in key bond lengths in poly(ANTH-yne) and the potential energy surface are provided in Figures S6 and S7 in the Supporting Information.

The bandgaps of poly(HEPT-yne) (*n* = 7) and poly(NONA-yne) (*n* = 9) are relatively larger and were therefore not included in the strain study. However, poly(BISANTH-yne), illustrated in Figure 5c–d has a low bandgap of 0.26 eV, with a Q ground state. An analogous polymer, poly(PERIPENT-yne), shown in Figure S13 in the Supporting Information, has also a Q ground state with and d_2_ = 1.37 Å and E_g_ = 0.79 eV. The bandgap for the latter is relatively larger for achieving a topological phase transition by realistic strains. Not surprisingly, however, poly(BISANTH-yne) indeed displays a topological Q to A phase transition close to ε = 4.2% strain, as shown in Figure 5c.

We conducted calculations to ascertain the topological phase in poly(BISANTH-yne) also using an extended finite chain model and verified the topological phase transition between nontrivial and trivial state based on the existence or absence of edge states (see Figure S12) in full analogy with the behavior of poly(BISANTH-yne) under strain. This decrease of E_g_ is attributed to the presence of vertical nodes in the polymer’s lowest unoccupied level, which is stabilized during stretching and the gap disappears at ∼4.2% strain, at the HOMO–LUMO level crossing as illustrated in Figure S8 in the Supporting Information. The recurrence of the V-shaped pattern in the BLA versus E_g_ plot, similar to that observed in the case of poly(PENT-yne) and poly(ANTH-yne), indicates the generality of the observed level crossing as a function of strain with possible venues for inducing topological phase transition in these and similar polymers by mechanical strain.

### 2.4 Suppression of the Peierls Distortion

**Figure 6:**
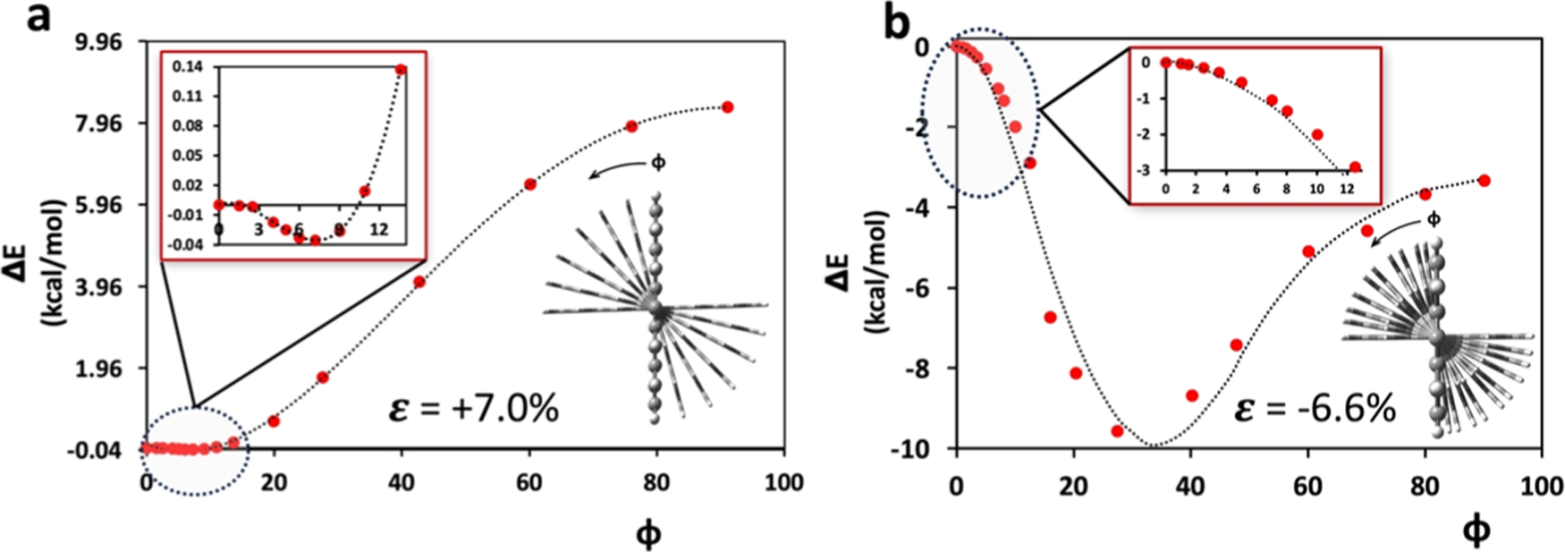
Relative energy gain as a function of out-of-plane inter-ring torsion, Φ. (a) Poly(PENT-yne) and (b) poly(ANTH-yne) based on out-of-plane symmetry breaking.

According to Peierls’ theorem, near E_g_ = 0, i.e., near the strain values where the level crossing occurs, there should be a vibrational mode that is strongly coupled to the gap such that the polymer would distort by lowering its energy and increasing E_g_ to a nonzero value. We now show that this is also indeed the case for the presented polymers. The mode that is most effective in raising the gap is an out-of-plane torsion, Φ, that reduces π-electron conjugation and leads to localization of the π-electronic structures to the regions of the fused rings and the triple bonds. To test this hypothesis, we performed computations by varying the out-of-plane inter-ring torsions as illustrated in Figure 6. To allow for this symmetry reduction, the unit cells were doubled in the computations.

Indeed, in all cases, we obtained an energy gain as a function of the symmetry-breaking torsion, Φ, which is defined in Figure S14. The magnitude of this energy gain is strongly system-dependent, as expected: Peierls’ theorem is silent about the magnitude of the energy gain due to the symmetry breaking. When exposed to a similar angle of distortion, poly(ANTH-yne) exhibits a greater energy relaxation compared to poly(PENT-yne). This disparity can be attributed to the compressed nature of poly(ANTH-yne) and the stretched state of poly(PENT-yne) at its topological phase transition point. The compression experienced by poly(ANTH-yne) makes it more susceptible to distortion as a means of alleviating steric crowding, unlike poly(PENT-yne). However, comparing the bandgaps under similar torsional distortions within 10° (*cf*. Figure S15 in the Supporting Information), both polymers exhibit similar magnitudes of bandgap opening.

It is important to observe that these gaps generated by the symmetry breaking due to nonzero torsion are minuscule on the order of 10 meV. Computationally such small gaps are challenging to evaluate accurately and to compare. Nevertheless, the key point in the presented results is that this Peierls energy gain within the polymer is small during full contact with the Au surface, which stabilizes the coplanar conformation. We are not able to perform a quantitative comparison, but there is no question that the energy gain generated by the symmetry breaking of poly(PENT-yne) amounts to about 0.04 kcal/mol, an amount that pales in comparison with any van der Waals stabilization by the Au surface. The case of poly(ANTH-yne) indicates an energy gain of about 10 kcal/mol, which overall might be competitive with the van der Waals stabilization by the Au surface. As stated above, this issue is system-dependent and will require further studies.

Nevertheless, we argue that at least for some of the systems where the energy gain by symmetry breaking is small, these systems lose their strict one-dimensionality due to the surface-polymer interaction allowing the Peierls distortion to be suppressed although not necessarily eliminated, completely. Note that the computation of E_g_ (see Figure S15 in the Supporting Information) as a function of Φ gives small but clearly nonzero values that increase with Φ, in agreement with Peierls’ theorem. The fact that the gap and energy gain values at small distortions are small allows the Peierls distortion at least partially to be suppressed by intermolecular interactions in this case.

### 2.5 Gap Reduction by Substitution

**Figure 7:**
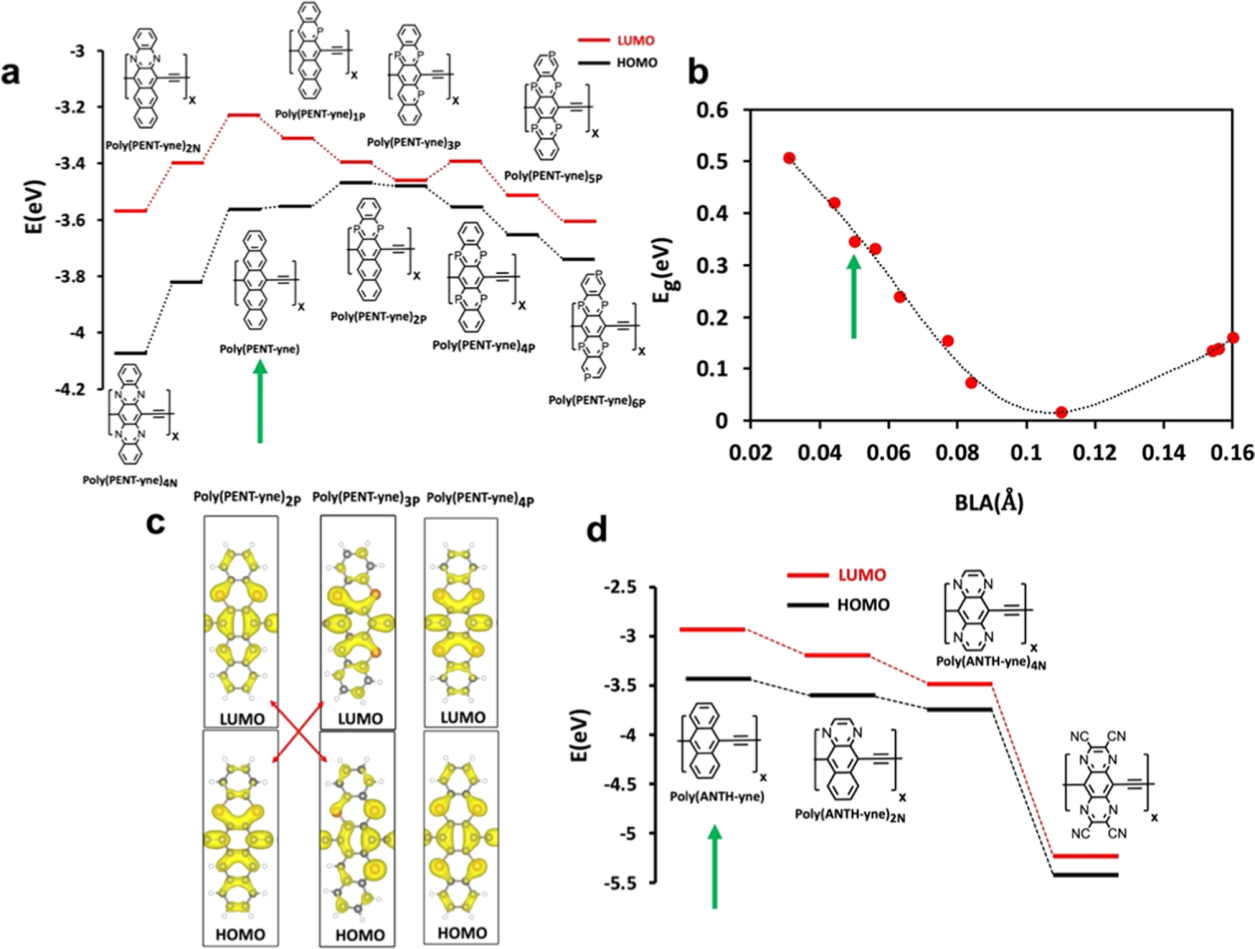
Effect of heteroatom substitution in π-conjugated ethynylene bridged [*n*]acene polymers. (a) Series of poly(PENT-yne) polymers with their HOMO and LUMO levels. (b) BLA values and the respective E_g_ values for the series shown in (a). (c) HOMO and LUMO densities of the unit cells near the level crossing. (d) Series of poly(ANTH-yne) polymers with their HOMO and LUMO levels. The green arrow indicates the unsubstituted polymer.

Chemical modification is a well-known method for altering the electronic properties and bandgaps of conjugated polymers. We employed two approaches to poly(PENT-yne) and poly(ANTH-yne): (I) replacing carbon atoms in the aromatic ring with nitrogen, and phosphorus atoms; and (II) introducing electron-donating or -withdrawing functional groups. These modifications and their effects on the bandgap are summarized in Figure 7 (*cf*. Supporting Information Table S1). The number and type of heteroatoms are denoted by a subscript of the names identifying each polymer. A few modifications that are less effective in reducing E_g_ are shown in Figure S17 in the Supporting Information for completeness.

The most effective modifications to achieve an energy gap reduction are illustrated in the series shown in Figure 7a. Starting with the unsubstituted poly(PENT-yne), a quinoid structure, it turns out that nitrogen substitution further stabilizes the quinoid structure with an increased E_g_, and as shown in Figure 7b, a decreased BLA (more pronounced quinoid structure). However, a series of phosphorus substitutions moved the structure on the BLA scale closer to aromatic structures, proceeding through a level crossing near poly(PENT-yne)_3P_. Beyond three P atom substitutions, the structure becomes more and more aromatic with increasing BLA and E_g_ values. Notably, for poly(PENT-yne)_3P_, the bandgap was nearly zero (E_g_ = 0.02 eV). This series illustrates the useful insight obtained by looking at substitutions as discrete sets of steps across the A/Q phase transition.

Doping by nitrogen atoms into poly(ANTH-yne) reduced the bandgap, as seen in Figure 7d. Replacing two carbon atoms with nitrogen results in a decrease of the gap from 0.49 to 0.39 eV, while incorporating another two nitrogen atoms further reduces it to 0.24 eV representing a 50% reduction compared to the parent poly(ANTH-yne) system. Additional four cyano groups generate a further decrease to 0. 19 eV, but the aromatic to quinoid transition has not been reached, although the BLA moved from 0.14 Å for poly(ANTH-yne) to 0.11 Å for poly(ANTH-yne)_4N+4CN_ in the quinoid direction.

These results established that suitable chemical perturbation can significantly lower the E_g_ value of π-conjugated polymers and even induce a topological transition through an orbital level crossing mechanism. By analyzing finite chains of poly(PENT-yne)_4N_ and poly(PENT-yne)_4P_ positioned at opposite sides of the topological phase transition (see Figure S18), we reinforced our stance on poly(PENT-yne)’s transition via chemical modification. The presence of edge states in poly(PENT-yne)_4N_ affirms its nontrivial nature (*Z*_2_ = 1), contrasting with poly(PENT-yne)_4P_, which lacks such states (*Z*_2_ = 0), further solidifying our argument.

## 3 Conclusions

Cirera et al.^17^ and González-Herrero et al.^18^ firmly established the existence of two topological phases (aromatic vs quinoid) of π-conjugated polymers on a gold surface but did not offer a route for a continuous phase transition because the phases were controlled by chemical composition, which by its very nature is *discrete*. We have discovered by means of density functional theory (DFT) computations that the phase transition can be achieved by applying *continuously* varied longitudinal strain. Accordingly, the bandgap can be tuned to minimal values that become zero at the phase boundary through a HOMO–LUMO level crossing. This level crossing is accomplished by stretching for poly(PENT-yne) and compressing for poly(ANTH-yne), in concordance with the fact that the former is an aromatic type and the latter is a quinoid type at zero strain. The mechanical strain affects different bonds differently, providing a mechanism for this continuous phase transition. Controlling strain can provide a novel route to achieve near-zero bandgap, provided the polymer is close to the aromatic/quinoid topological transition point. Additionally, chemical modification offers a viable method to manipulate the bandgap and move the electronic structure from one side of the topological transition toward the other. We also show that the Au surface template serves as a suppressor of the symmetry-breaking Peierls torsional distortion that would otherwise intervene for zero-bandgap 1D polymers. The suppression of the Peierls distortion may not be complete, but the enforcing of coplanarity of the repeat units robs the Peierls distortion driving force from its most efficient energy-lowering mechanism in this case, which is an interunit torsional distortion.

## 4 Methods

Density functional theory (DFT) was used with the Quantum Espresso program for all calculations.^27^ The Perdew, Burke, and Ernzerhof (GGA-PBE) generalized gradient approximation functional^28^ in conjunction with Vanderbilt ultrasoft pseudopotentials was used.^29^ The plane-wave basis set was limited to the kinetic energy cutoff of 140 Ry for wave functions and 1400 Ry for charge density. A smearing width of 0.02 Ry was applied using the Marzari-Vanderbilt smearing method.^30^ For Brillouin zone integration in self-consistent field (SCF) calculations, a *k*-mesh of 12 × 1 × 1 was used.^31^ The k-point grid was further increased to 18 × 1 × 1 for band structure calculations. Grimme’s DFT-D2 empirical formalism was employed to account for dispersion interactions.^32^ The energy convergence threshold was set to 10^–8^ Ry, and the total force in the geometry optimizations was less than 0.001 au for all calculations. Energy gaps appear at the edge of the Brillouin zone at *k* = π/*a* except for doubled unit cells where the gaps are at *k* = 0 as dictated by symmetry. Unless specified otherwise, orbital electron densities are plotted using VESTA software with an isovalue set at 0.0008.^33^ In studying topological phase transitions within a finite polymer chain containing 15 acene units placed in a large box, a γ *k*-point and a reduced energy cutoff of 30 Ry were applied to ensure computational feasibility. The supercells included ample space using a unit cell of 20 Å in both directions perpendicular to the polymer chain.

To apply longitudinal strain to the polymers, we compressed and stretched them by adjusting the length of the lattice vector, ***a⃗***. The percentage of applied strain ε(%) is defined as


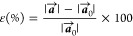
1Here, ***a⃗***_0_ is the optimized equilibrium lattice vector at zero strain and ***a⃗*** is the adjusted lattice vector chosen to impose strain in the system; the negative value corresponds to compression. The other two lattice parameters were chosen such that the interchain interactions are negligible. Structures were reoptimized at each fixed strain value.

## Supporting Information Available

Energy band structures; orbital diagrams; BLA scheme; structural parameters; potential energy surfaces; partial density of states (PDOS) and edge states formation; Young’s modulus; and optimized coordinates with lattice vectors (PDF)
