# Supplementary material for: Continuous Topological Transition and Bandgap Tuning in Ethynylene-Linked Acene π-Conjugated Polymers through Mechanical Strain
Source: Chem Mater. 2024 Jan 31;36(3):1395–404. doi: 10.1021/acs.chemmater.3c02547 (PMC10876101; doi:10.1021/acs.chemmater.3c02547)
Supplement: Supplementary file 1 — cm3c02547_si_001.pdf [file cm3c02547_si_001.pdf]

Supporting Information

Continuous Topological Transition and Bandgap Tuning in Ethynylene-Linked Acene π-Conjugated Polymers through Mechanical Strain

Rameswar Bhattacharjee*, Miklos Kertesz*

Department of Chemistry, Georgetown University, Washington, District of Columbia, 20007, United States.

Corresponding Authors

*__Miklos Kertesz__ - Department of Chemistry, Georgetown University, Washington, District of Columbia 20057, United States.

ORCID ID- orcid.org/0000-0002-7930-3260

Email: kertesz@georgetown.edu

***Rameswar Bhattacharjee** − Department of Chemistry, Georgetown University, Washington, District of Columbia 20057, United States.

ORCID ID- orcid.org/0000-0002-6631-5991

Email: rb1820@georgetown.edu

Table of contents

1. Band structures, (Figures S1-S3).
2. Orbitals, BLA, and band gap of poly(PENT-yne), (Figures S4,S5).
3. Changes in bond distances vs strain, potential energy surface of poly(ANTH-yne), (Figures S6,S7).
4. HOMO, LUMO plots, potential energy surfaces, optimized unit cells, BLA and band gap of poly(BISANTH-yne), (Figures S8-S11).
5. Supplementary Note S1| Zero energy edge states
   Topological phase transition and topological invariant calculation for poly(BISANTH-yne) , Figure S12.
6. Optimized unit cell, HOMO, and LUMO of poly(PERIPENT-yne), (Figure S13).
7. Definition of dihedral angle and Bandgap vs dihedral plot for poly(PENT-yne) and poly(ANTH-yne), (Figures S14, S15).
8. Supplementary Note S2| Effects of substitutional doping
9. Optimized unit cells, optimized cell vectors, and relevant bond distances of doped pentacene polymers, Figure S16.
10. HOMO and LUMO energy levels and bandgaps of unsubstituted poly(PENT-yne) and its doped analogs, Supplementary Table S1.
11. HOMO LUMO levels for the functionalized poly(PENT-yne), Figure S17.
12. Topological phase transition and topological invariant calculation for Poly(PENT-yne)$_{4N}$ and Poly(PENT-yne)$_{4P}$, Figure S18.
13. Supplementary Note S3| Young modulus (Y).
14. Supplementary Table S2 | Calculated Young Modulus (Y) data.
15. Supplementary Table S3 | Optimized Coordinates and lattice vectors.

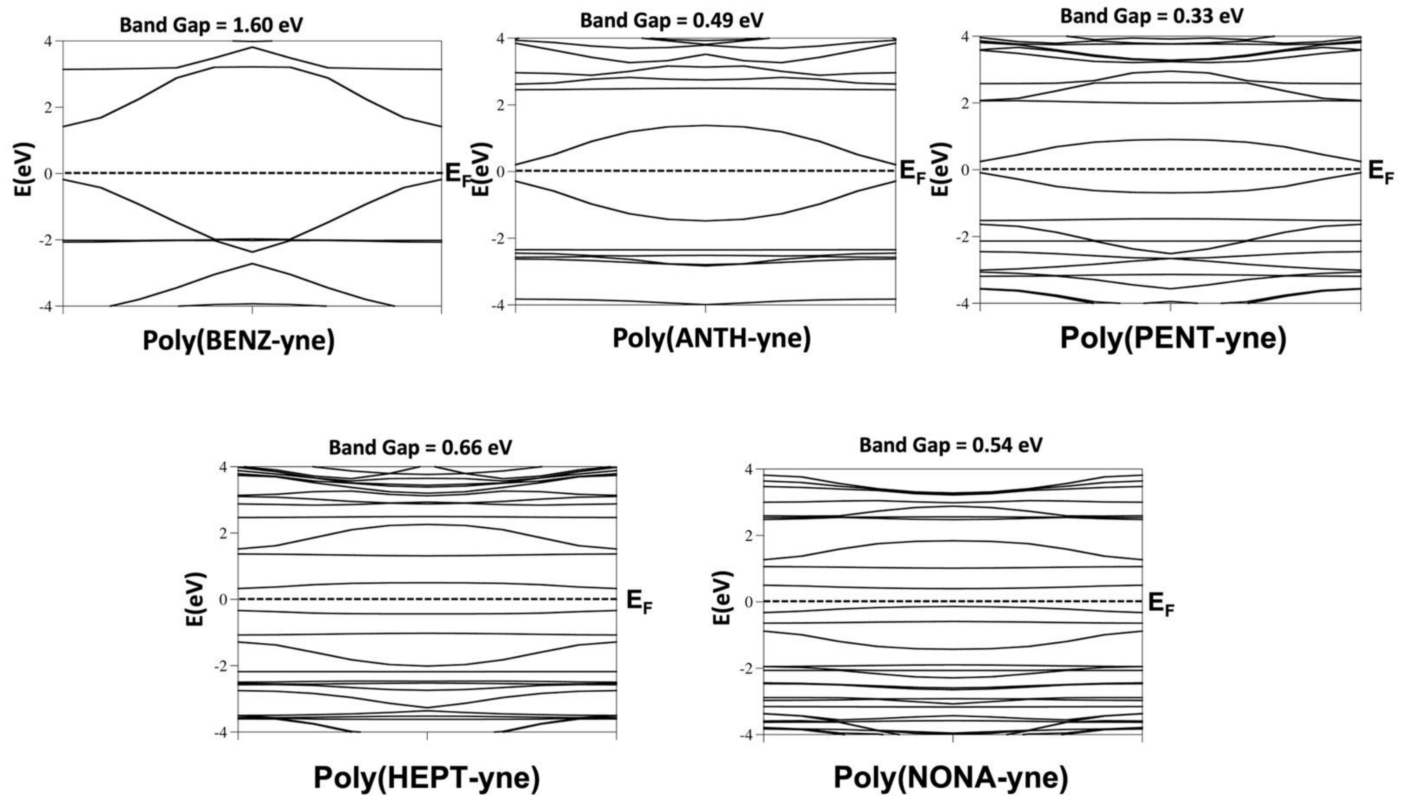

Figure S1 | Band structure plot for all the single-stranded polymers discussed in the manuscript.

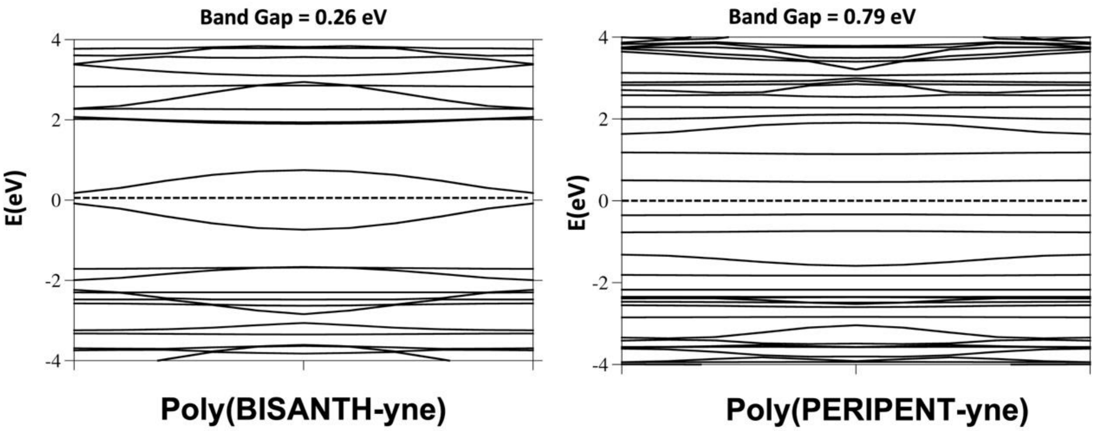

Figure S2 | Band structure for two peri-acene-based polymers discussed in the manuscript.

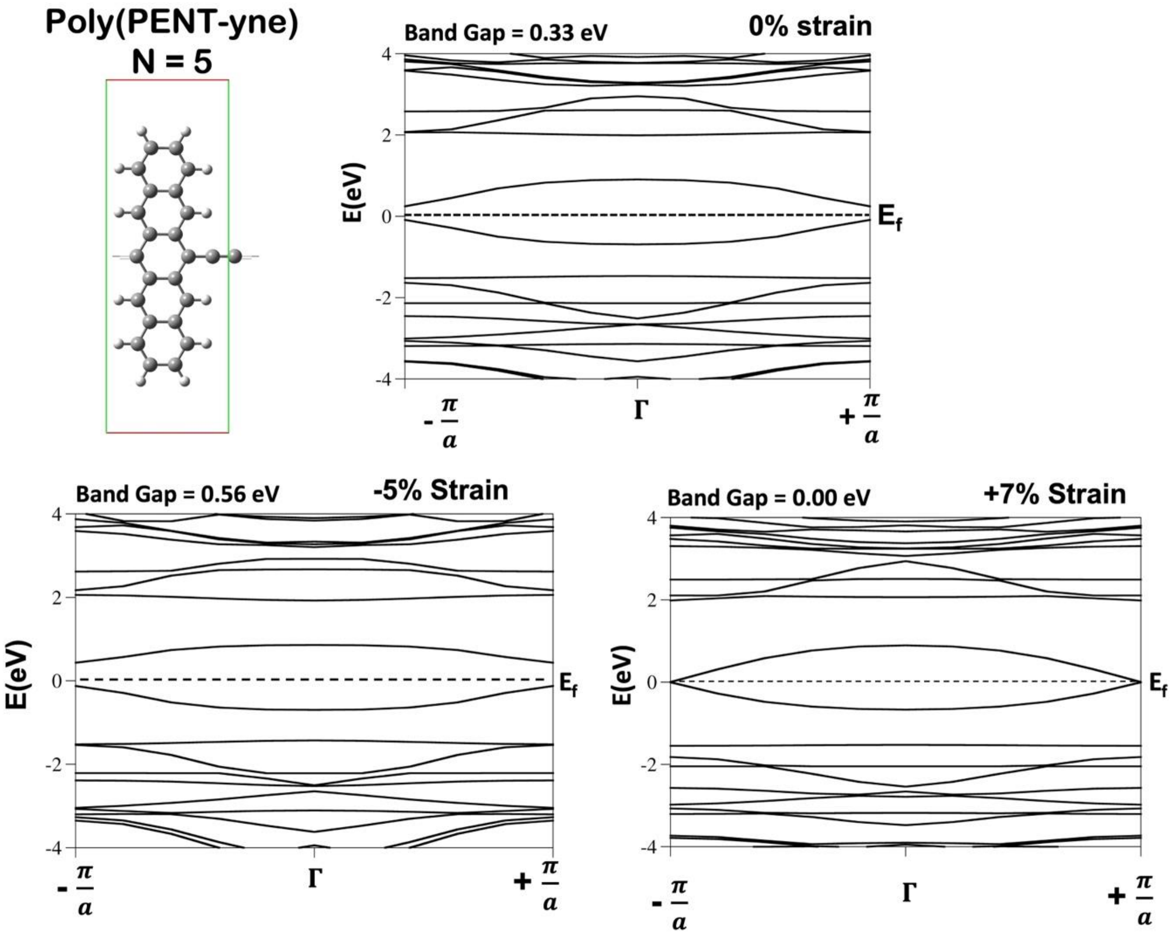

Figure S3 | Band structure plot of pentacene based polymer at 0% -5% and +7% of applied strain.

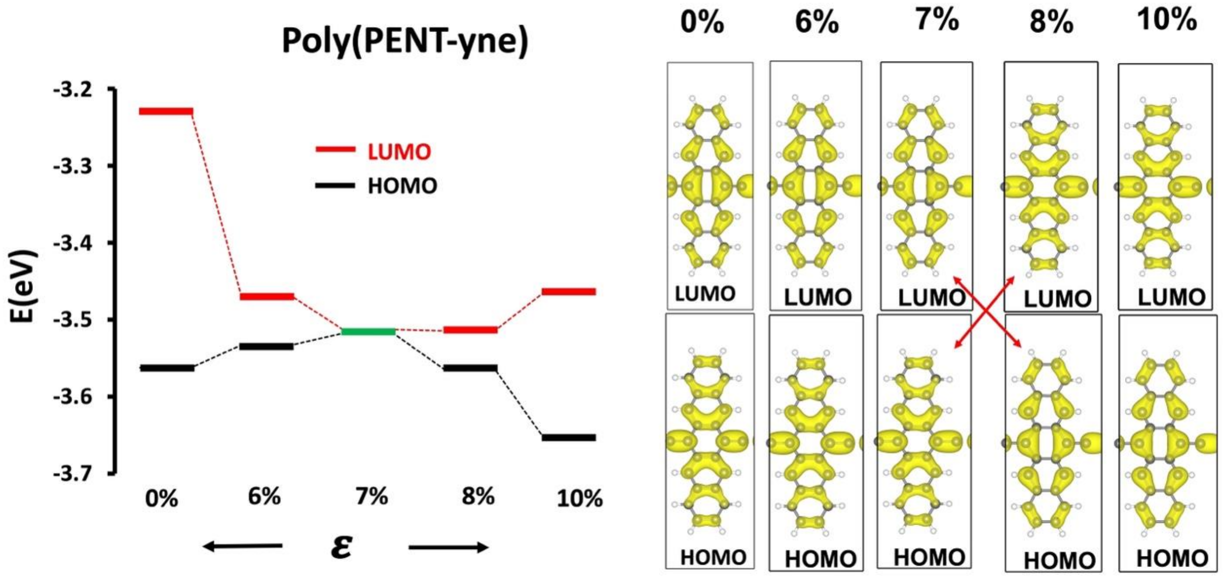

Figure S4 | The energy levels of the polymer were analyzed at different applied strains of 0%, 6%, 7%, and 8%. The highest occupied molecular orbital (HOMO) and lowest unoccupied molecular orbital (LUMO) were identified for each strain. Notably, a zero-band gap was observed at 7% stretched pentacene, which is indicated by a green bar in the analysis near where the HOMO-LUMO level crossing occurs. The electron density of the HOMO and LUMO is shown on the right.

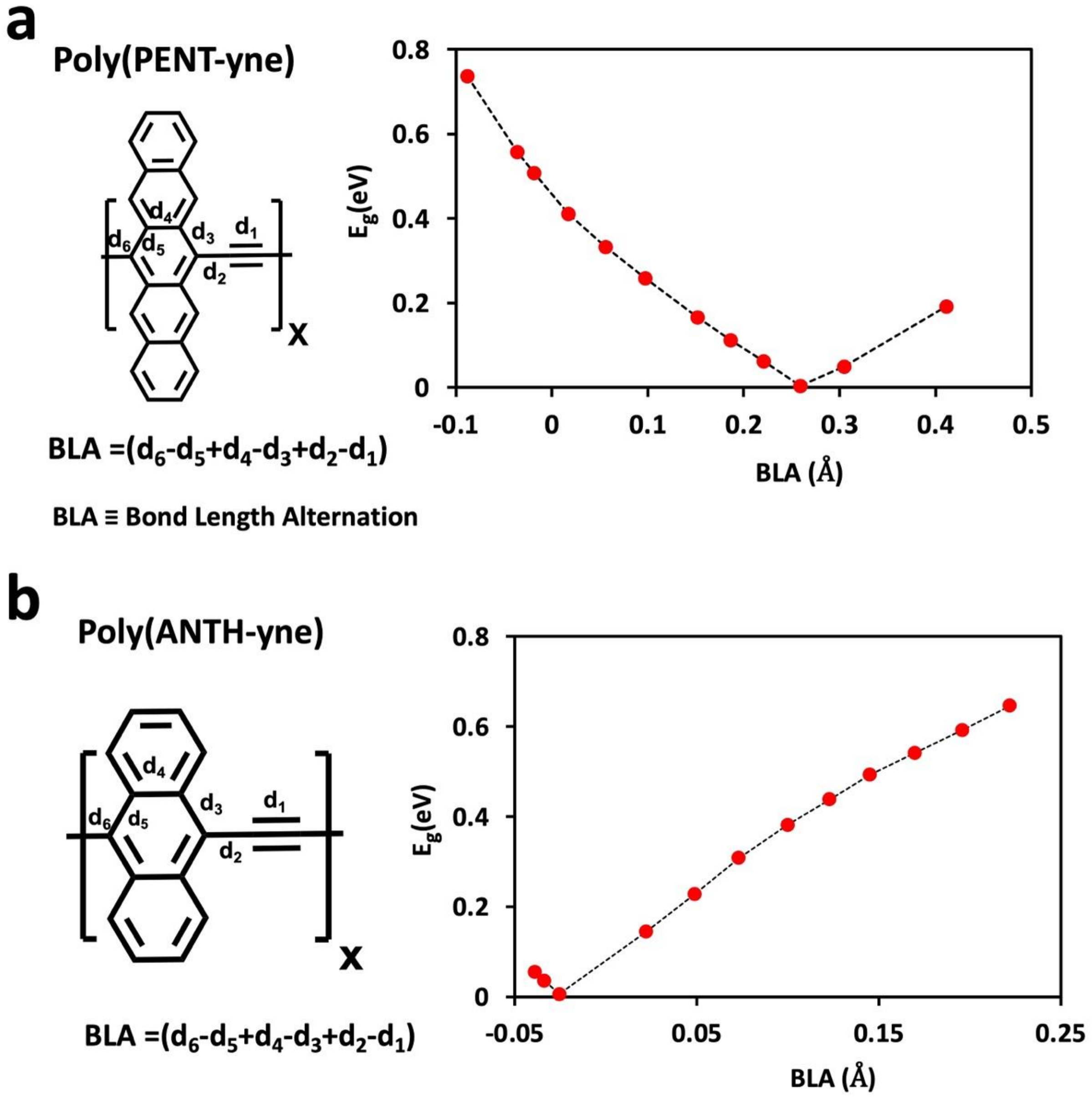

Figure S5 | (a)Left: definition of bond length alternation (BLA) of poly(PENT-yne). Right: Bandgap, E_g, of poly(PENT-yne) as a function of BLA as obtained at different levels of strain.

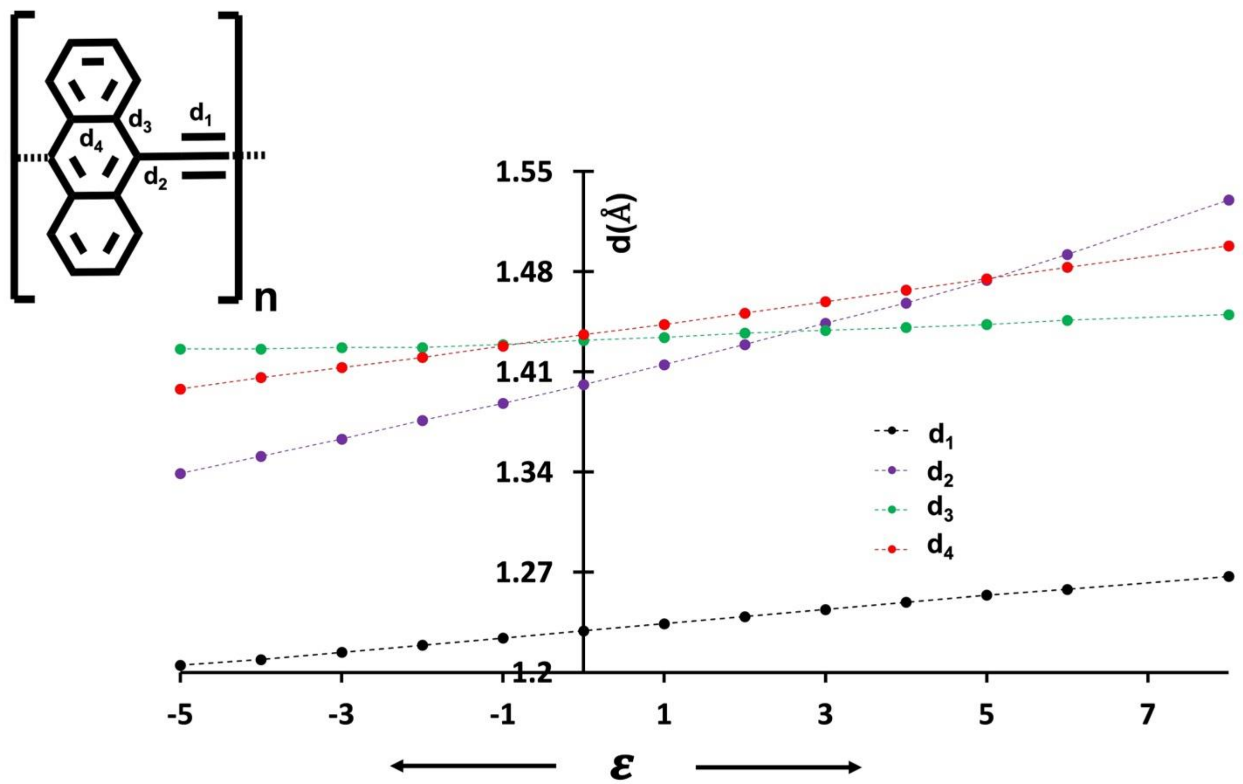

Figure S6 | Chemical formula of the unit cell of poly(ANTH-yne) and the changes in four relevant bond lengths (d_1, d_2, d_3, d_4) as a function of strain.

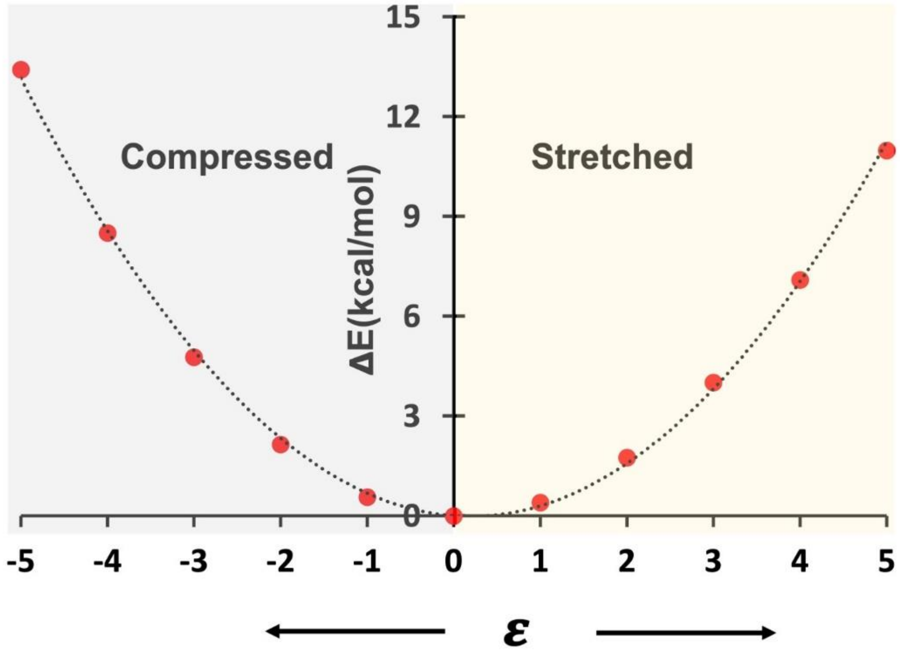

Figure S7 | Total energy as a function of strain of poly(ANTH-yne).

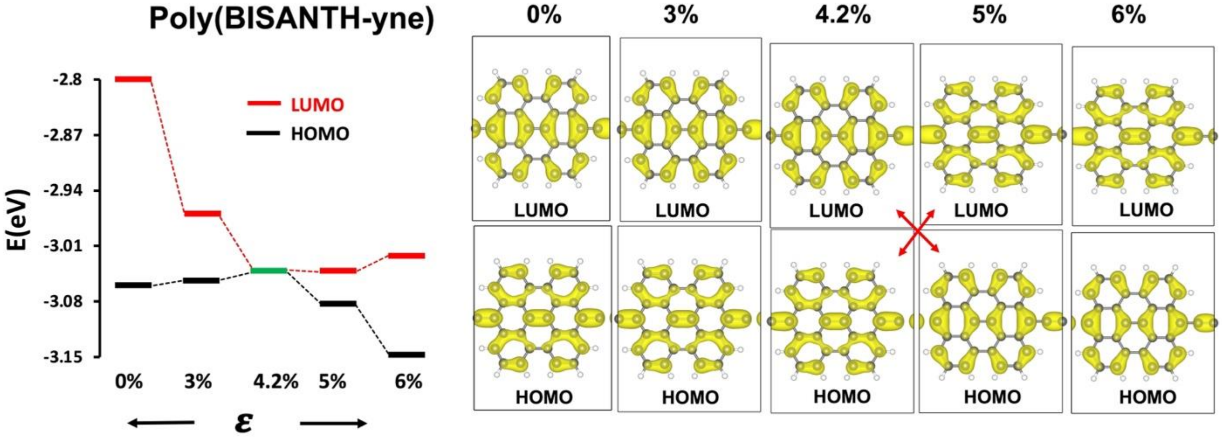

Figure S8 | HOMO and LUMO levels of poly(BISANTH-yne) at select strain values. The orbital level crossing is indicated on the right showing the orbital electron densities.

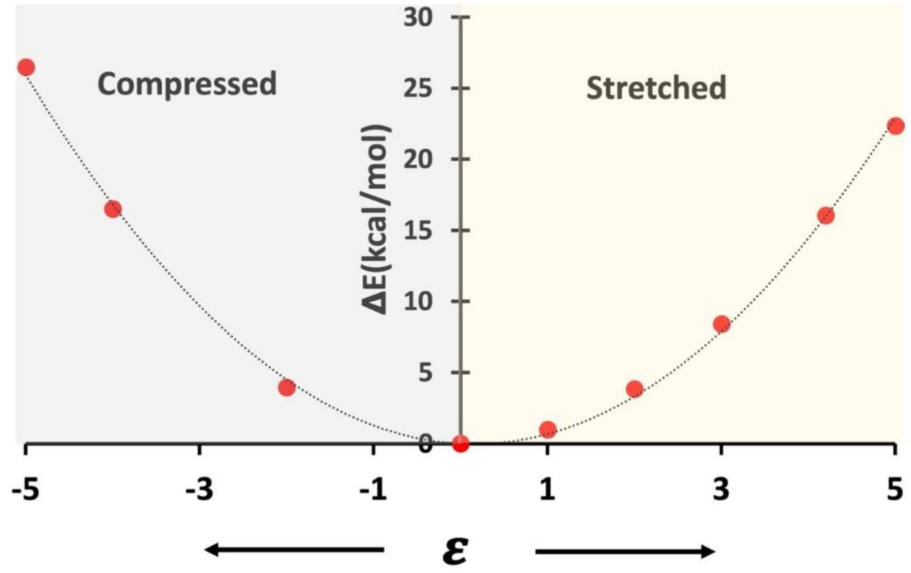

Figure S9 | Total energy as a function of the strain of poly(BISANTH-yne).

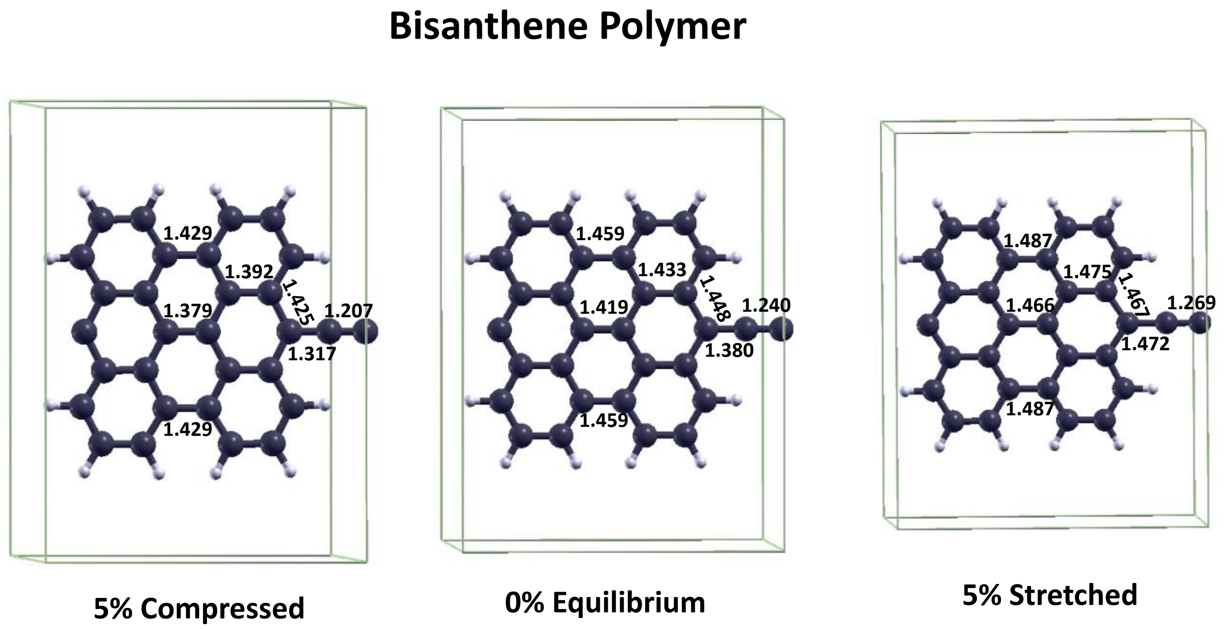

Figure S10 | Optimized unit cell of poly(BISANTH-yne) at -5%, 0%, and 5% of applied strain. Selected bond lengths (in Å) are shown. Color code: Carbon atoms are in dark grey, and hydrogen atoms are in white.

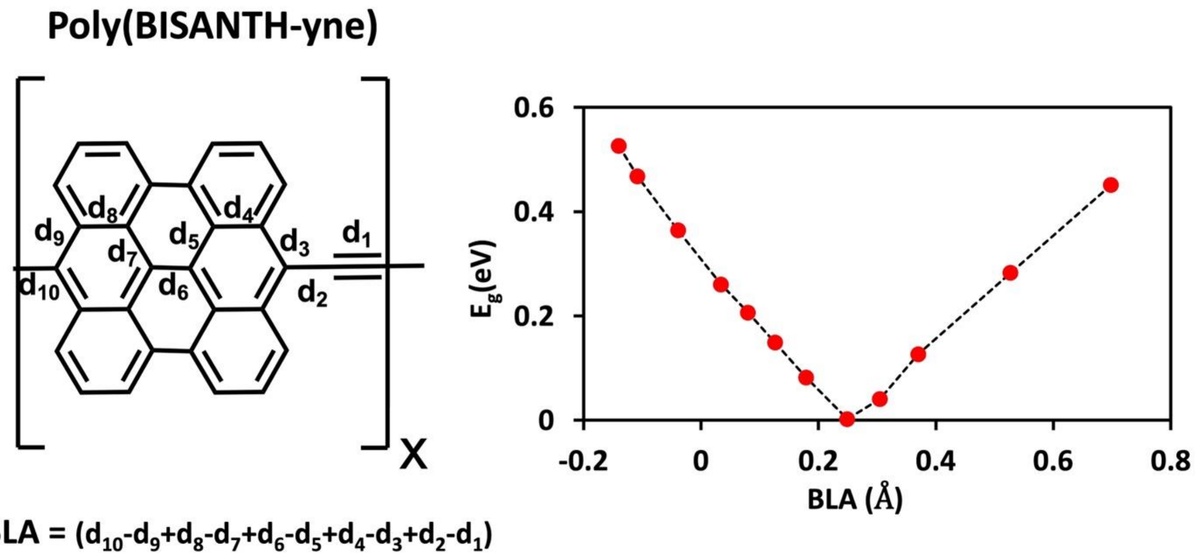

Figure S11 | Definition of the bond length alteranation (BLA) of poly(BISANTH-yne), left, and band gap as a fucntin of BLA, right.

Supplementary Note 1| Zero energy edge states

In our investigation of the long finite chain model for poly(BISANTH-yne), comprising 15 bisanthene units, we followed a similar approach to the poly(PENT-yne). We constructed two model chains representing poly(BISANTH-yne) at strain levels of 0% and 8%, positioned on either side of the topological phase boundary, delineating two distinct topological phase regions. This process follows the one described in the main text for poly(PENT-yne).

As depicted in Figure S12a-S12b, we observed a conspicuous presence of a doubly degenerate zero-energy edge state in the system at the unstressed state, thereby categorizing it as non-trivial topological state (Z_2=1). However, at 8% tensile strain, there was a notable absence of any edge state formation, leading to the characterization of the system at ε=8% as being in a trivial phase(Z_2=0). Note that “zero energy” refers to the energy of the state relative to the Fermi level.

This critical analysis underscores a pivotal observation: in poly(BISANTH-yne), a continuous topological phase transition occurs under mechanical strain, affirming the dynamic nature of its topological properties across varying strain levels.

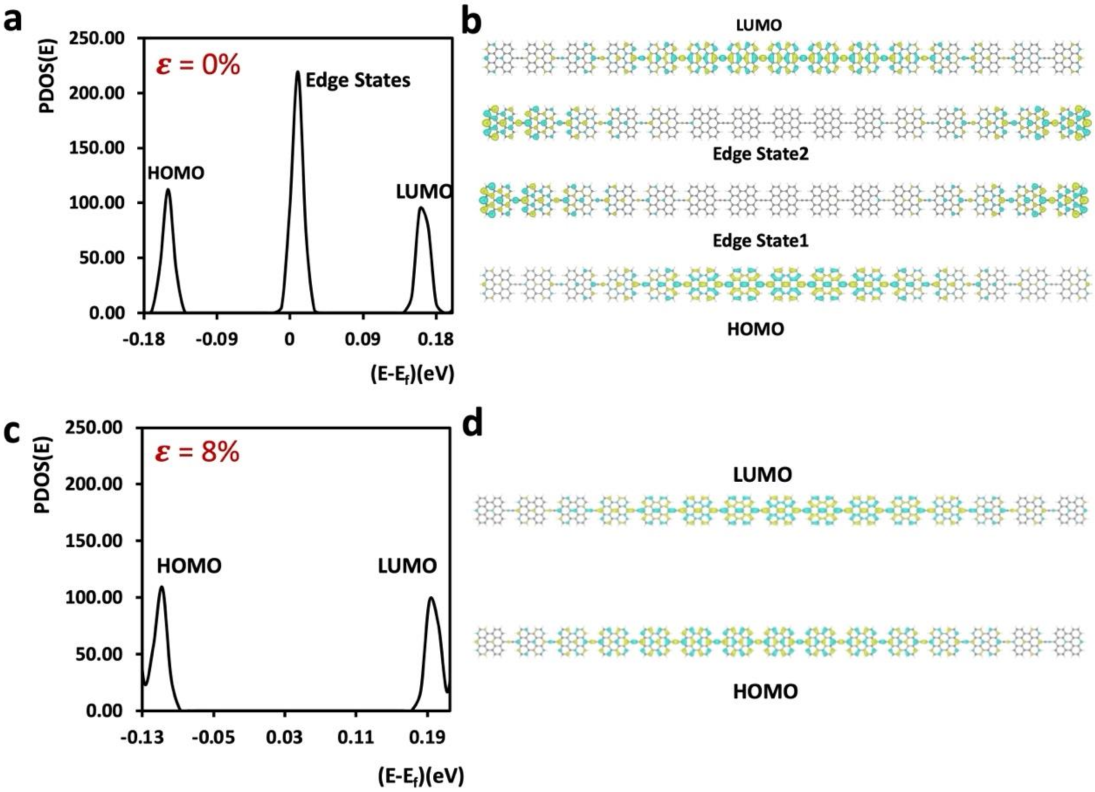

Figure S12 | Topological phase transition and topological invariant **a** Computed Projected Density of States (PDOS) for the finite H-terminated poly(BISANTH-yne) chain comprising 15 units extracted from the optimized periodic structure of poly(BISANTH-yne) without strain. **b** Frontier molecular orbitals (isovalue= 5×10^{-5}) for four bands (edge states are nearly degenerate) depicted in Figure S12a. **c** PDOS presentation of a comparable finite H-terminated poly(BISANTH-yne) chain extracted from the periodic structure of poly(BISANTH-yne) optimized at 8% tensile strain. **d** Frontier molecular orbitals of the corresponding to Figure S12c.

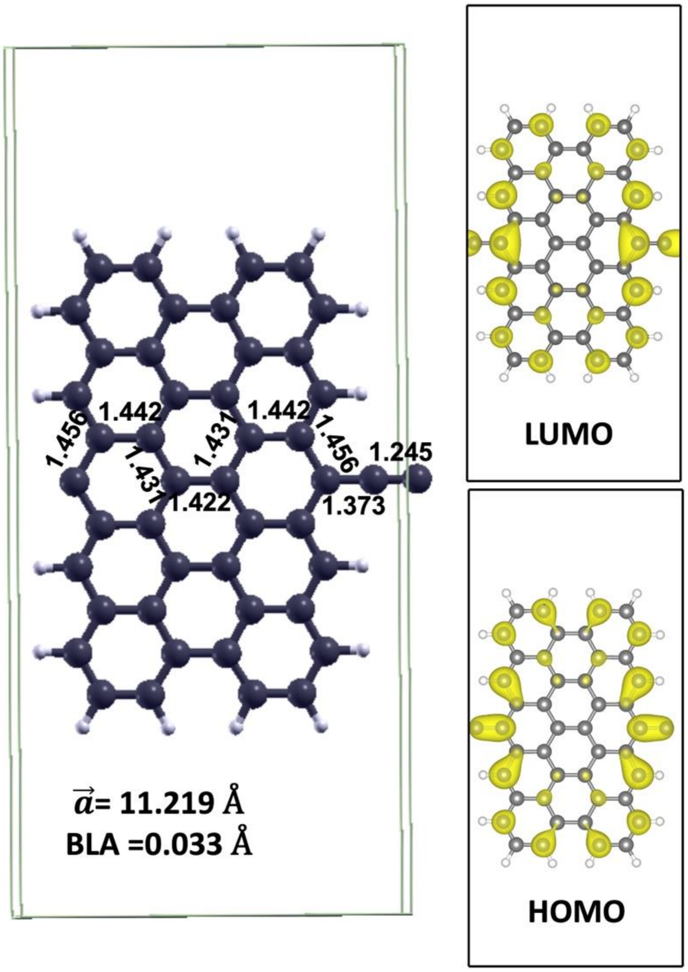

Figure S13 | Optimized unit cell of poly(PERIPENT-yne). HOMO and LUMO are shown at the right at k=0.0

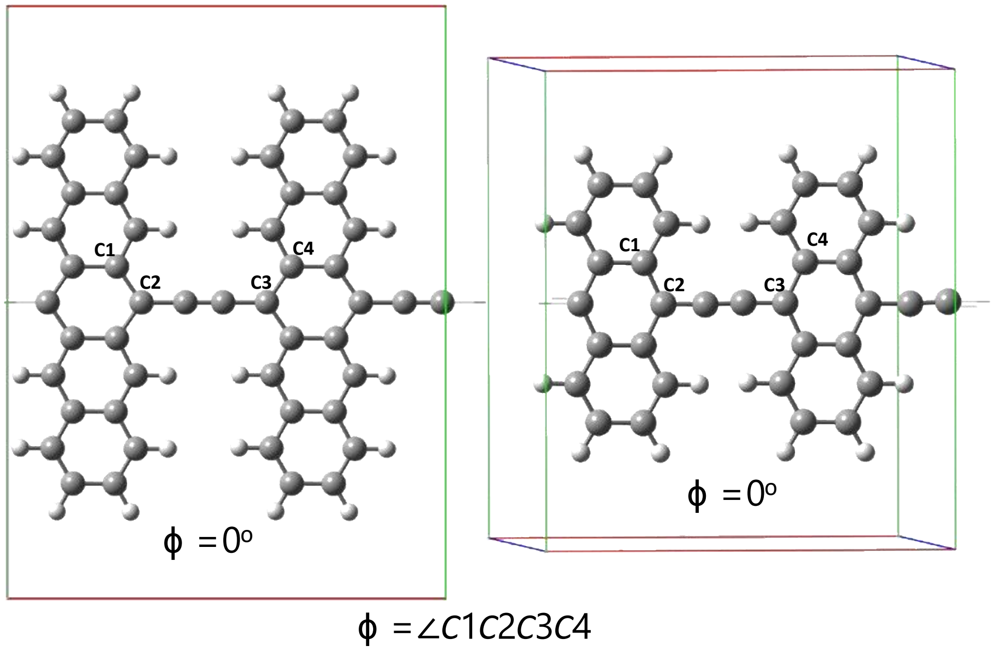

Figure S14 | Definition of the dihedral angle(**ϕ**) used the function to compute energies in a doubled unit-cell (2×1×1 supercell)

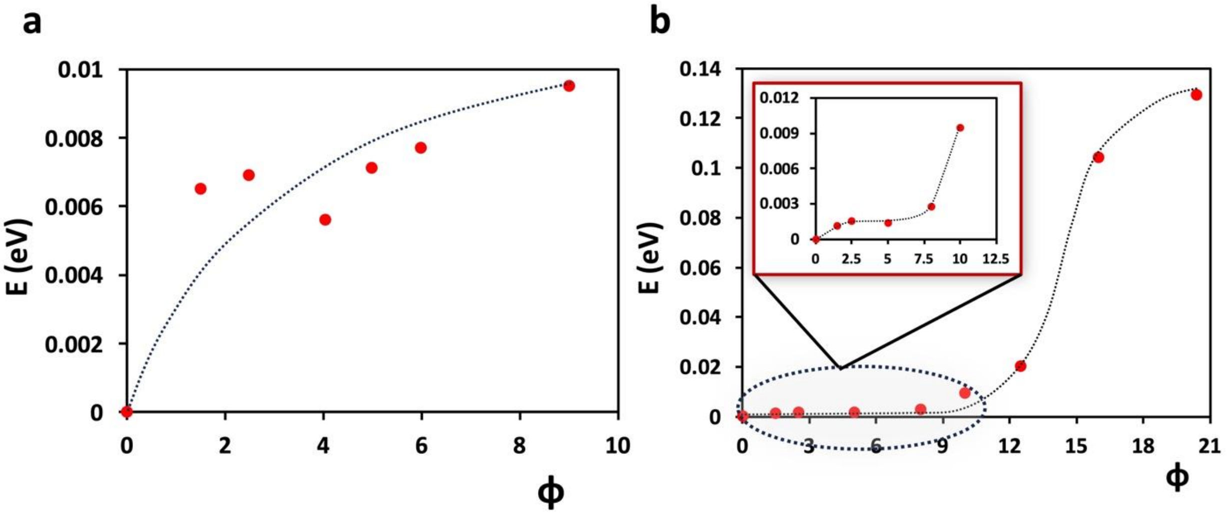

Figure S15 | Band gap (E_g) vs dihedral angle (**ϕ**) plot for **a,** poly(PENT-yne) and **b,** poly(ANTH-yne)

Supplementary Note S2| Effects of substitutional doping

Initially we tried to replace carbon atoms with nitrogen since nitrogen is the most appropriate substitute for carbon in aromatic rings, as reported earlier.[1, 2] However, we discovered that substituting carbon with nitrogen in poly(PENT-yne) resulted in an increase in the band gap instead of reducing it. We also incorporated phosphaacene units into our study by systematically introducing phosphorus atoms into poly(PENT-yne) to observe their impact on its optical properties. Phosphorus as a doping atom received significant attention after the successful exfoliation of phosphorene, which resembles graphene and has high charge carrier mobility.[3] We selected three phosphaacenes from a previous study[4] and used them as the aromatic units of ethyne-linked 1D polymers.

To investigate the influence of functional groups, we selected some electron-withdrawing groups and methyl as a donating group in the pentacene unit. Our findings indicate that the installation of functional groups had a nearly equal impact on the HOMO and LUMO, resulting only in a slight change in the band gap. The smallest band gap of 0.24 eV was observed when four fluorine atoms were attached to each terminal of the pentacene unit. For the other functional groups such as -Br, -CN, and -Me, a similar effect on the band gaps of poly(PENT-yne) was observed. It is important to note that axial functionalization, in this case, was not effective in reducing the band gap; rather, it led to an increase in the band gap (E_g). A pictorial representation of the effects of various functional groups on the energy level of the HOMO and LUMO is provided Fig. S16.

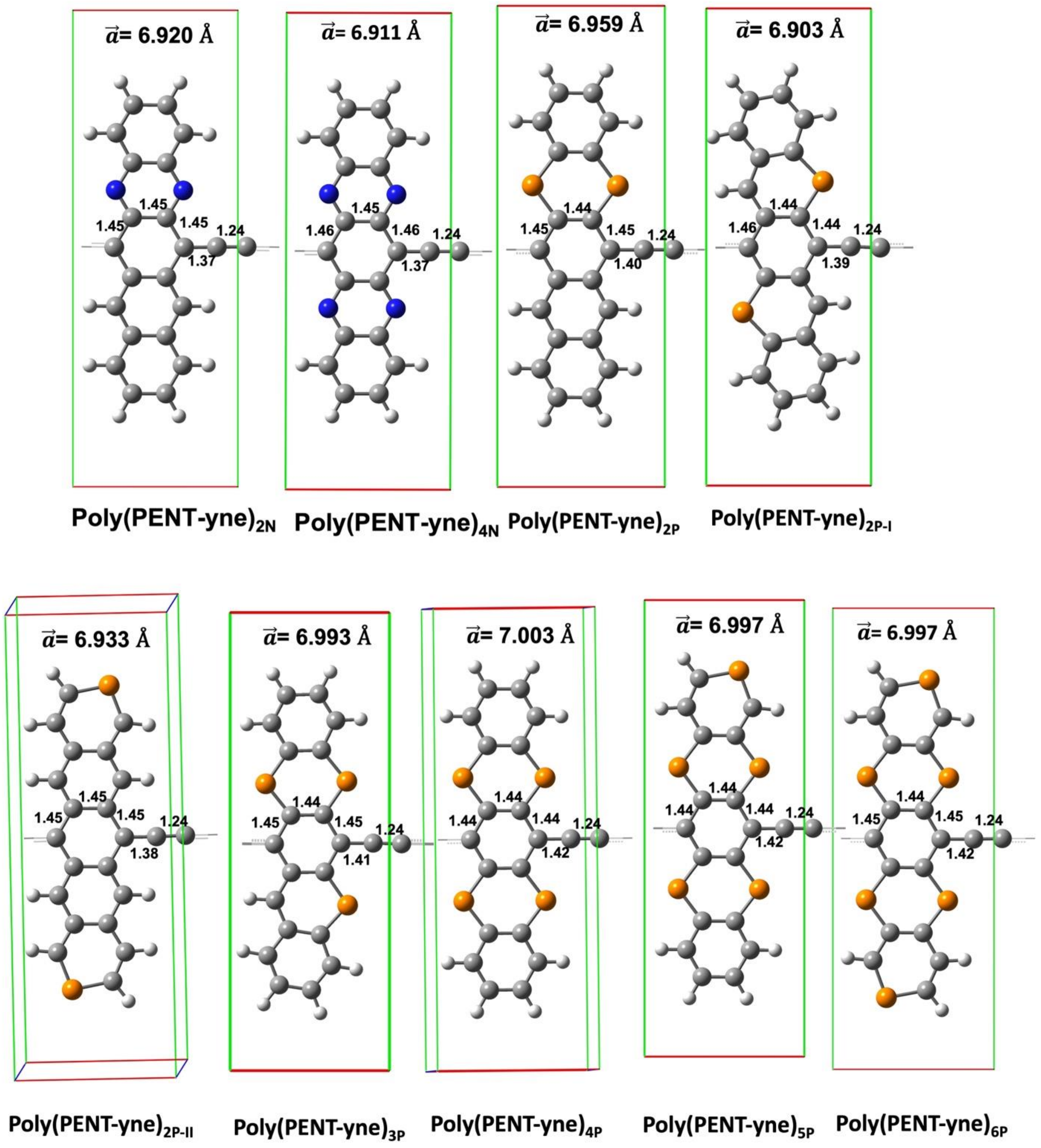

Figure S16| Optimized unit cells of all the chemically doped pentacene poly(PENT-yne) are presented. Key bond distances are provided in Å. Color code: carbon is grey, nitrogen is blue, hydrogens are white, and phosphorous is saffron.

Supplementary Table S1 | HOMO and LUMO energy levels and bandgaps of unsubstituted poly(PENT-yne) and its doped analogs. The structural diagrams show aromatic structures regardless of whether the ground state structure is on the aromatic or quinonoid side of the transition.

**Unit cell diagram**	**Name indicating number of substitution sites**<br>Ground state is aromatic (A) or quinonoid (Q)	**HOMO (eV)**	**LUMO (eV)**	**E_g (eV)**
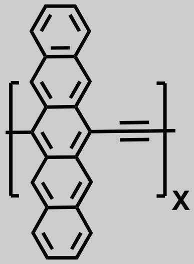	Poly(PENT-yne)<br>Q	-3.56	-3.23	0.33
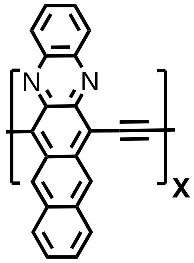	Poly(PENT-yne)$_{2N}$<br>Q	-3.82	-3.40	0.42
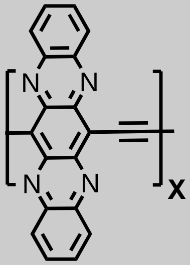	Poly(PENT-yne)$_{4N}$<br>Q	-4.07	-3.56	0.51

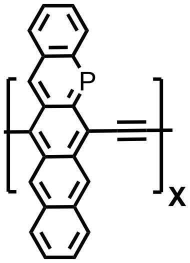	Poly(PENT-yne)$_{1P}$<br><br>Q	-3.55	-3.31	0.24
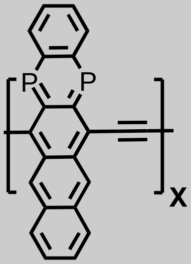	Poly(PENT-yne)$_{2P}$<br><br>Q	-3.46	-3.39	0.07
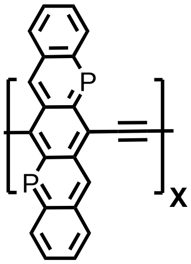	Poly(PENT-yne)$_{2P-I}$<br><br>Q	-3.34	-3.39	0.15
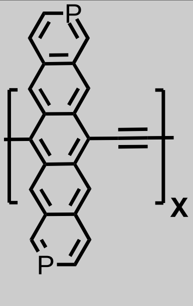	Poly(PENT-yne)$_{2P-II}$<br><br>Q	-3.82	-3.48	0.34
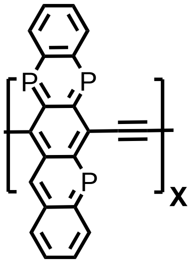	Poly(PENT-yne)$_{3P}$<br><br>A	-3.48	-3.46	0.02

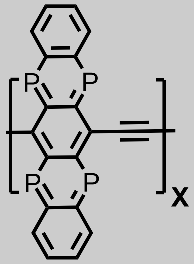	Poly(PENT-yne)$_{4P}$<br><br>A	-3.55	-3.39	0.16
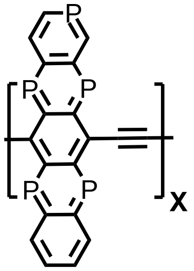	Poly(PENT-yne)$_{5P}$<br><br>A	-3.65	-3.51	0.14
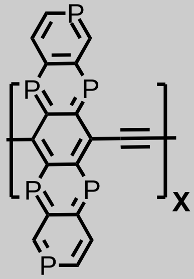	Poly(PENT-yne)$_{6P}$<br><br>A	-3.74	-3.60	0.13

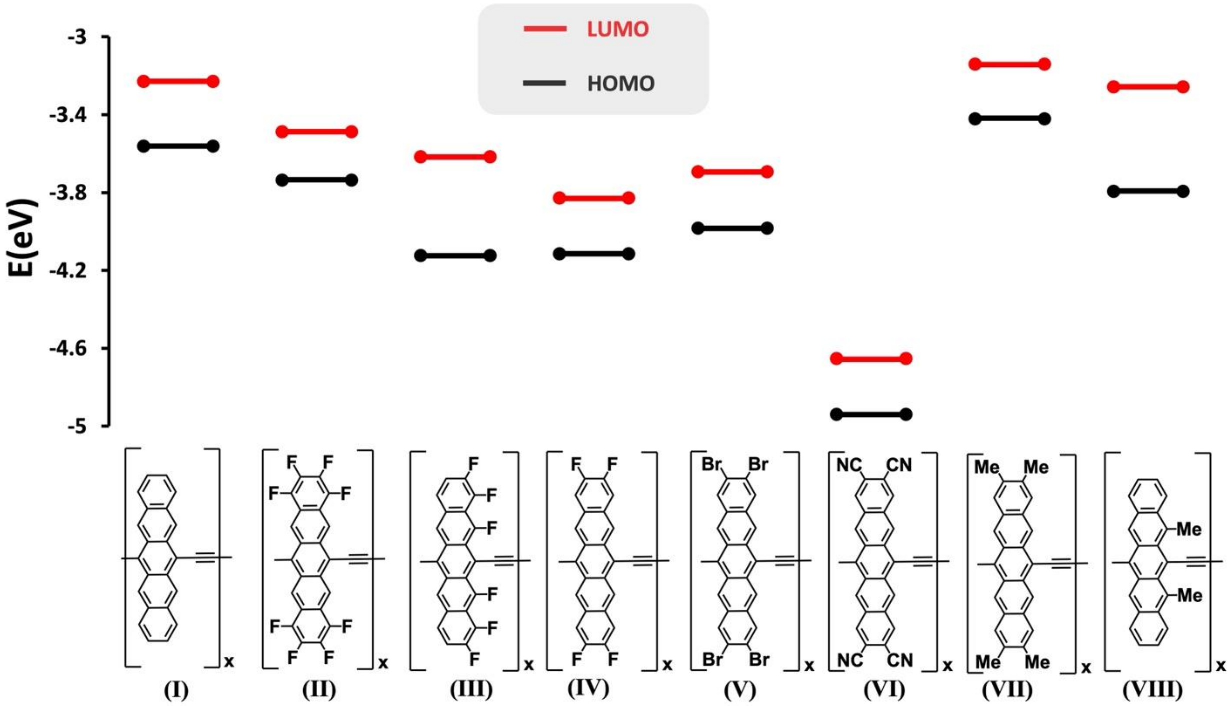

Figure S17| Frontier HOMO and LUMO orbital energy levels of selected functionalized poly(PENT-yne) derivatives.

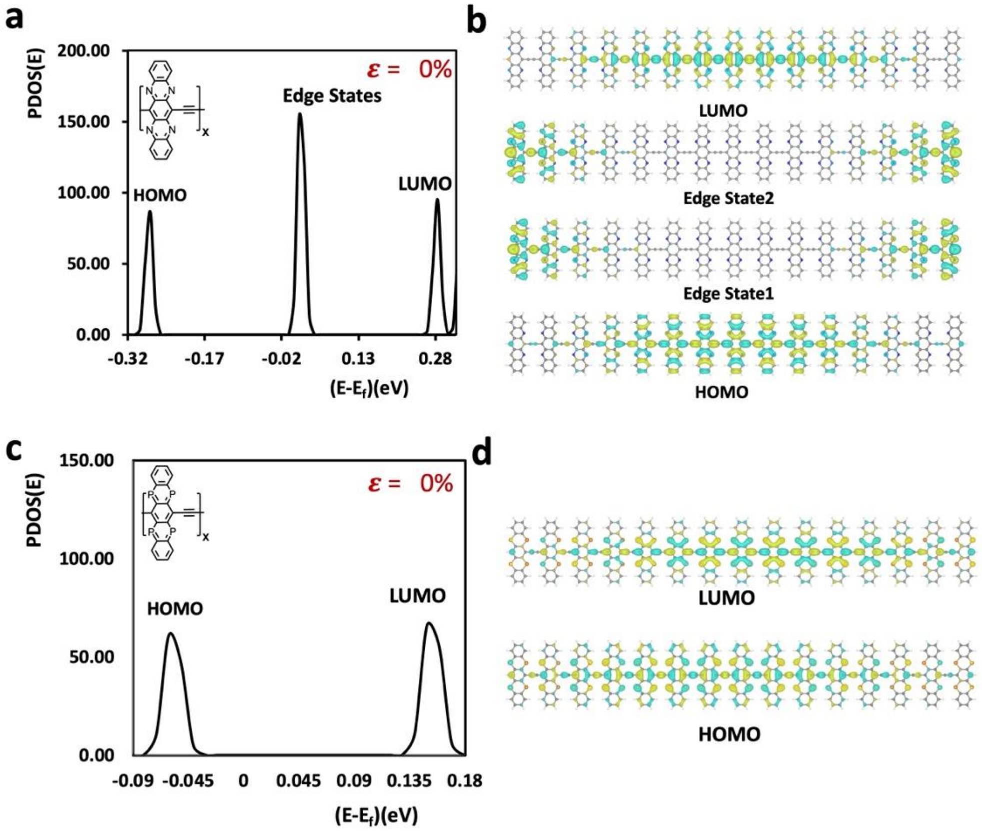

Figure S18 | Topological phase transition and topological invariant **a** Computed Projected Density of States (PDOS) for the finite H-terminated poly Poly(PENT-yne)$_{4N}$ chain comprising 15 units extracted from the optimized periodic structure of Poly(PENT-yne)$_{4N}$. **b** Frontier molecular orbitals (isovalue= 5×10^{-5}) for four bands (edge states are nearly degenerate) are depicted in Figure S18a. **c** PDOS presentation of a comparable finite H-terminated Poly(PENT-yne)$_{4P}$ chain extracted from the optimized periodic structure of Poly(PENT-yne)$_{4P}$. **d** Frontier molecular orbitals of corresponding to Figure S18c.

Supplementary Note S3| Young modulus (Y)

The Young modulus (Y) is determined by taking the second derivative of the total energy of the systems across the polymer's cross-section:[5]

$$Y = \frac{1}{A}\left(\frac{d^2E}{d\varepsilon^2}\right) \tag{3}$$

where A is the cross-section of the polymer. E is the per unit cell total energy. Note that the cross-section area is very appropriate. The calculated value of Young's modulus, assuming A=48Å^2, for poly(PENT-yne) is 113 Gpa. This is reasonable if compared to polyacetylene (PA), poly(methyleneoxide) (PMO), polyethylene (PE), and other organic polymers indicating the overall consistency of the methods used.[6-9] Additionally, we calculated Y for two other polymers namely poly(ANTH-yne) and poly(BISANTH-yne) are provided in Table S2 below.

Supplementary Table S2 | Calculated Young Modulus (Y) for the three most relevant polymers discussed. The cross-section values used for calculating Y are also provided.

**Unit Cell Diagram**	**Cross Section (Å^2)**	**Young modulus (GPa)**
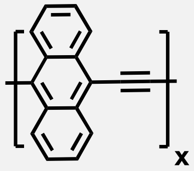 **Poly(ANTH-yne)**	31	156
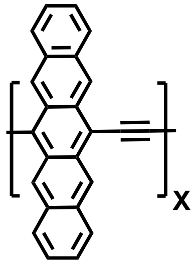 **Poly(PENT-yne)**	48	113
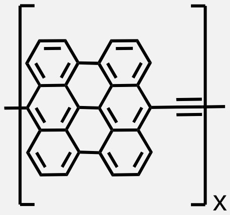 **Poly(BISANTH-yne)**	31	191

Supplementary Table S3 | Optimized Coordinates and the respective optimized lattice vector lengths $|\vec{a}|$:

Benzene polymer:

Poly(BENZ-yne) (n=1):

Lattice vector $|\vec{a}|$ =6.879605626 Å

C	3.7469966656	6.2856876235	7.5000000000
C	2.3619136839	6.2916049322	7.5000000000
C	4.4755292696	7.4972935815	7.5000000000
C	1.6418300390	7.5086626767	7.5000000000
C	2.3704901906	8.7204283448	7.5000000000
C	3.7556098987	8.7142234814	7.5000000000
C	5.8875030008	7.4976106602	7.5000000000
C	7.1092716539	7.5084125715	7.5000000000
H	4.3098169237	9.6513484545	7.5000000000
H	1.8272145437	9.6639225327	7.5000000000
H	4.2912294532	5.3425102400	7.5000000000
H	1.8086137865	5.3541131611	7.5000000000

Anthracene Polymer and its Derivates:

Poly(ANTH-yne) (n=3):

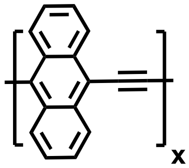

Lattice vector $|\vec{a}|$ =6.911356486 Å

C	3.7392957466	3.8222289252	7.5000000000
C	4.4348416574	5.0082754487	7.5000000000
C	3.7682190082	6.2622051790	7.5000000000
C	2.3318895743	6.2714586163	7.5000000000
C	1.6468933073	5.0278969954	7.5000000000
C	2.3245541307	3.8317012155	7.5000000000
C	4.4970885398	7.4942188486	7.5000000000
C	1.6167737378	7.5117675369	7.5000000000
C	2.3457750132	8.7438635937	7.5000000000
C	3.7821534688	8.7345732557	7.5000000000
C	4.4675378798	9.9780863236	7.5000000000
H	5.5549416526	9.9635533130	7.5000000000

C	3.7903566836	11.1744262459	7.5000000000
C	2.3755945912	11.1839624690	7.5000000000
C	1.6796104242	9.9979511525	7.5000000000
H	4.2803818731	2.8762440272	7.5000000000
H	5.5225843690	5.0046112409	7.5000000000
H	0.5595159577	5.0420782405	7.5000000000
H	1.7685599933	2.8944195829	7.5000000000
H	4.3465622805	12.1117757937	7.5000000000
H	1.8346565576	12.1299220994	7.5000000000
H	0.5918871600	10.0019479622	7.5000000000
C	5.8981547469	7.4930609732	7.5000000000
C	7.1270248551	7.5125358912	7.5000000000

Poly(ANTH-yne)$_{2N}$:

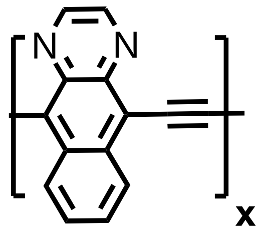

Lattice vector $|\vec{a}|$ = 6.913998539Å

C	3.7430305518	3.8584596398	7.5000000000
C	4.4382184347	5.0457643147	7.5000000000
C	3.7706304999	6.2985304702	7.5000000000
C	2.3339775793	6.3072950110	7.5000000000
C	1.6496153531	5.0638858860	7.5000000000
C	2.3283147604	3.8676637381	7.5000000000
C	4.5056572728	7.5289808532	7.5000000000
C	1.6111520738	7.5453220441	7.5000000000
C	2.3470322084	8.7715419936	7.5000000000
C	3.7820988983	8.7626201315	7.5000000000
C	3.7906486554	11.0492368320	7.5000000000
C	2.3699005436	11.0585655618	7.5000000000
H	4.2815405077	2.9108463414	7.5000000000
H	5.5256899642	5.0473407867	7.5000000000
H	0.5623395003	5.0820040410	7.5000000000
H	1.7763072952	2.9277035309	7.5000000000
H	4.3529149825	11.9884097596	7.5000000000

H	1.8215390274	12.0057268649	7.5000000000
C	5.9017603131	7.5451600712	7.5000000000
C	7.1291388769	7.5626628577	7.5000000000
N	4.4870403670	9.9305286405	7.5000000000
N	1.6578179569	9.9490143001	7.5000000000

Poly(ANTH-yne)$_{4N}$:

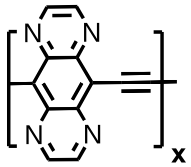

Lattice vector $|\vec{a}|$ = 6.907284634 Å

C	3.7433569827	3.9805832980	7.5000000000
C	3.7664409937	6.2678819187	7.5000000000
C	2.3321100408	6.2768127502	7.5000000000
C	2.3242342283	3.9897118050	7.5000000000
C	4.5082642468	7.4948027935	7.5000000000
C	1.6024435567	7.5112527900	7.5000000000
C	2.3443055774	8.7381801936	7.5000000000
C	3.7786374860	8.7292391873	7.5000000000
C	3.7867174449	11.0163481969	7.5000000000
C	2.3675956708	11.0254872558	7.5000000000
H	4.2939302844	3.0350701065	7.5000000000
H	1.7597235072	3.0524147293	7.5000000000
H	4.3512924339	11.9536126668	7.5000000000
H	1.8170770152	11.9710255142	7.5000000000
C	5.8959057633	7.4943264292	7.5000000000
C	7.1220843784	7.5117073367	7.5000000000
N	4.4821840604	9.8959792722	7.5000000000
N	1.6564495946	9.9146099369	7.5000000000
N	4.4544081307	5.0915278393	7.5000000000
N	1.6286672287	5.1100001701	7.5000000000

Poly(ANTH-yne)$_{4N}$-4CN-substituted Anthracene:

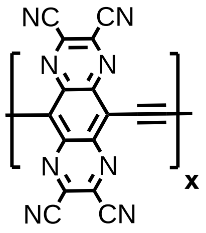

Lattice vector $|\vec{\boldsymbol{a}}|$ = 6.927309603 Å

C	3.8593539991	6.4740420972	10.0000000000
C	3.8709678518	8.7672337250	10.0000000000
C	2.4332791221	8.7757939825	10.0000000000
C	2.4157360410	6.4828218966	10.0000000000
C	4.6188706998	9.9921221370	10.0000000000
C	1.6970816474	10.0078668587	10.0000000000
C	2.4449723293	11.2327952003	10.0000000000
C	3.8826745489	11.2241872140	10.0000000000
C	3.9003779417	13.5171619852	10.0000000000
C	2.4567547340	13.5260410670	10.0000000000
C	6.0075253707	9.9914122228	10.0000000000
C	7.2357036069	10.0084416637	10.0000000000
N	4.5833274666	12.3851121561	10.0000000000
N	1.7588945740	12.4025488348	10.0000000000
N	4.5571139329	7.5973915125	10.0000000000
N	1.7326790754	7.6149780472	10.0000000000
C	4.6079398170	14.7601040797	10.0000000000
N	5.1054034383	15.8146326637	10.0000000000
C	1.7691568575	14.7796293214	10.0000000000
N	1.2962732447	15.8456621165	10.0000000000
C	4.5471118486	5.2202538556	10.0000000000
N	5.0203182976	4.1546551264	10.0000000000
C	1.7083161360	5.2400270325	10.0000000000
N	1.2112772156	4.1851333036	10.0000000000

Pentacene Polymers and its derivatives:

Poly(PENT-yne) (n=5):

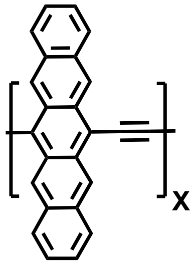

Lattice vector $|\vec{a}|$ = 6.933462797 Å

C	3.9052971517	6.2934366946	10.0000000000
C	4.5767049325	7.5224577433	10.0000000000
C	3.9096165084	8.7469820066	10.0000000000
C	2.4626917774	8.7497125224	10.0000000000
C	1.7944337897	7.5236931303	10.0000000000
C	2.4663015051	6.2928645866	10.0000000000
C	4.6549782141	9.9935935723	10.0000000000
C	1.7254464126	10.0039029453	10.0000000000
C	2.4718800698	11.2526740513	10.0000000000
C	3.9185864155	11.2454470111	10.0000000000
C	4.5966150793	12.4643874032	10.0000000000
H	5.6852037529	12.4509653353	10.0000000000
C	3.9356768103	13.6992079121	10.0000000000
C	2.4962026250	13.7106468471	10.0000000000
C	1.8135548013	12.4844644088	10.0000000000
H	5.6654660042	7.5248382881	10.0000000000
H	0.7057624918	7.5271808412	10.0000000000
H	0.7248888166	12.4899249850	10.0000000000
C	6.0348152611	9.9926763022	10.0000000000
C	7.2788028847	10.0053888301	10.0000000000
C	4.6347824318	14.9372811028	10.0000000000
C	3.9568103799	16.1333133162	10.0000000000
C	2.5365042306	16.1461624139	10.0000000000
C	1.8285448461	14.9666206517	10.0000000000
H	5.7232918421	14.9192891900	10.0000000000
H	4.5062055128	17.0749374610	10.0000000000
H	2.0081111439	17.0992758904	10.0000000000
H	0.7398596569	14.9807297571	10.0000000000
C	2.4865079531	3.8578957804	10.0000000000
C	3.9068101385	3.8594914552	10.0000000000
C	4.5935438384	5.0499445505	10.0000000000

C	1.7883503159	5.0428271531	10.0000000000
H	1.9505757673	2.9093560104	10.0000000000
H	4.4490943691	2.9136334459	10.0000000000
H	5.6818673483	5.0612470031	10.0000000000
H	0.6996936347	5.0395184013	10.0000000000

Poly(PENT-yne)$_{2N}$:

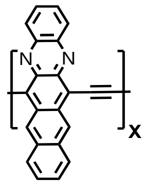

Lattice vector $|\vec{a}|$ = 6.920476649 Å

C	3.9139182361	6.3451059415	10.0000000000
C	4.5868537774	7.5755715021	10.0000000000
C	3.9195003296	8.7998653953	10.0000000000
C	2.4743132650	8.8033213847	10.0000000000
C	1.8045304496	7.5794608252	10.0000000000
C	2.4749828787	6.3466927226	10.0000000000
C	4.6660321060	10.0472201642	10.0000000000
C	1.7341334929	10.0572034297	10.0000000000
C	2.4786640662	11.3076695303	10.0000000000
C	3.9291909543	11.3000453573	10.0000000000
C	3.9393930391	13.5971120682	10.0000000000
C	2.5009034493	13.6071441708	10.0000000000
H	5.6748898698	7.5825086195	10.0000000000
H	0.7158162757	7.5884186645	10.0000000000
C	6.0393599239	10.0663528588	10.0000000000
C	7.2814455240	10.0758462549	10.0000000000
C	4.6423316122	14.8297874300	10.0000000000
C	3.9556234714	16.0211366480	10.0000000000
C	2.5341926209	16.0328300733	10.0000000000
C	1.8235686041	14.8548008993	10.0000000000
H	5.7277558061	14.7817578199	10.0000000000
H	4.5004954556	16.9653849674	10.0000000000
H	2.0075560550	16.9873473293	10.0000000000

H	0.7372419576	14.8350269844	10.0000000000
C	2.4868033737	3.9105539123	10.0000000000
C	3.9064948914	3.9104585811	10.0000000000
C	4.5982404352	5.0988779079	10.0000000000
C	1.7930710401	5.0986316883	10.0000000000
H	1.9485382587	2.9626958124	10.0000000000
H	4.4461085088	2.9630449483	10.0000000000
H	5.6867823366	5.1018472505	10.0000000000
H	0.7044269327	5.0971586700	10.0000000000
N	4.6271611014	12.4379665713	10.0000000000
N	1.7960110088	12.4562322867	10.0000000000

Poly(PENT-yne)$_{4N}$:

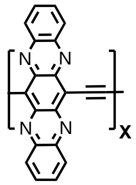

Lattice vector $|\vec{a}|$ = 6.911312829Å

C	3.9108607251	6.4481849097	10.0000000000
C	3.9189725406	8.7450063632	10.0000000000
C	2.4689511413	8.7484072787	10.0000000000
C	2.4718768633	6.4499750514	10.0000000000
C	4.6645832456	9.9944114112	10.0000000000
C	1.7291092105	10.0040110967	10.0000000000
C	2.4754384288	11.2554101227	10.0000000000
C	3.9254064024	11.2479875701	10.0000000000
C	3.9353903533	13.5442914059	10.0000000000
C	2.4965452350	13.5541702360	10.0000000000
C	6.0320682233	9.9944508941	10.0000000000
C	7.2729998870	10.0039858423	10.0000000000
C	4.6384082226	14.7769769763	10.0000000000
C	3.9507174304	15.9683224555	10.0000000000
C	2.5294531217	15.9798336638	10.0000000000
C	1.8183987232	14.8013635331	10.0000000000
H	5.7242672516	14.7316149562	10.0000000000
H	4.4959793661	16.9125972089	10.0000000000
H	2.0018353451	16.9340481528	10.0000000000

H	0.7317809908	14.7838760717	10.0000000000
C	2.4816651594	4.0246585364	10.0000000000
C	3.9031323669	4.0236936838	10.0000000000
C	4.6018765942	5.2087545020	10.0000000000
C	1.7820265276	5.2093837779	10.0000000000
H	1.9455554135	3.0750542260	10.0000000000
H	4.4398696907	3.0746424303	10.0000000000
H	5.6882283577	5.2419874669	10.0000000000
H	0.6954451765	5.2391355028	10.0000000000
N	4.6236404478	12.3848645937	10.0000000000
N	1.7917837625	12.4025732311	10.0000000000
N	4.6085105632	7.6024659985	10.0000000000
N	1.7766889233	7.6069203901	10.0000000000

Poly(PENT-yne)$_{1P}$:

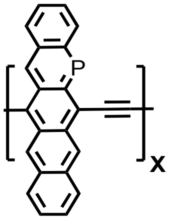

Lattice vector $|\vec{\boldsymbol{a}}|$ = 6.920545667 Å

C	4.1060466683	6.2514897264	10.0000000000
C	4.0880757830	8.7056047440	10.0000000000
C	2.6432961940	8.6955248327	10.0000000000
C	2.6664687865	6.2414627132	10.0000000000
C	4.8120678893	9.9647316031	10.0000000000
C	1.9008979628	9.9386731332	10.0000000000
C	2.6285609705	11.2026379277	10.0000000000
C	4.0721523599	11.2111938142	10.0000000000
C	3.8211621832	13.9511055914	10.0000000000
C	2.4077770645	13.6920867892	10.0000000000
C	6.1973611515	9.9622615902	10.0000000000
C	7.4382594128	9.9285836981	10.0000000000
C	4.2652254699	15.3035955556	10.0000000000
C	3.3685464084	16.3490728612	10.0000000000
C	1.9729542165	16.0926847105	10.0000000000
C	1.5081213408	14.7997647641	10.0000000000
H	5.3375571470	15.5078583119	10.0000000000
H	3.7296464922	17.3780989825	10.0000000000

H	1.2714209363	16.9269038998	10.0000000000
H	0.4395948451	14.5823062690	10.0000000000
C	2.6872204358	3.8072387292	10.0000000000
C	4.1082070865	3.8168486984	10.0000000000
C	4.7957617091	5.0070377397	10.0000000000
C	1.9895436859	4.9903879621	10.0000000000
H	2.1526137524	2.8574428929	10.0000000000
H	4.6506182619	2.8720441526	10.0000000000
H	5.8843097479	5.0143419212	10.0000000000
H	0.9014013680	4.9911279378	10.0000000000
P	5.0350249124	12.6832593967	10.0000000000
C	4.7682585811	7.4864082985	10.0000000000
C	1.8899554249	12.3889830527	10.0000000000
H	0.8031715178	12.3011971507	10.0000000000
H	5.8567027430	7.5011684809	10.0000000000
C	1.9840563348	7.4638823068	10.0000000000
H	0.8957747271	7.4549247016	10.0000000000

Poly(PENT-yne)$_{2P}$:

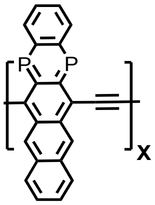

Lattice vector $|\vec{a}|$ = 6.959663779Å

C	3.9409623796	6.1549395206	10.0000000000
C	3.9511886149	8.6099476391	10.0000000000
C	2.5002542276	8.6135464718	10.0000000000
C	2.4992637450	6.1593070330	10.0000000000
C	4.6886467320	9.8553167117	10.0000000000
C	1.7684710136	9.8623033101	10.0000000000
C	2.5127256752	11.1110936747	10.0000000000
C	3.9497220897	11.1067996393	10.0000000000
C	3.9628382924	13.9235195404	10.0000000000
C	2.5314263114	13.9308214011	10.0000000000
C	6.0870063567	9.8577881075	10.0000000000
C	7.3296398832	9.8636916378	10.0000000000
C	4.6494335319	15.1755236708	10.0000000000
C	3.9713548786	16.3708639012	10.0000000000
C	2.5537433077	16.3785110384	10.0000000000

C	1.8604672915	15.1911155996	10.0000000000
H	5.7394095257	15.1679425384	10.0000000000
H	4.5222948832	17.3115071726	10.0000000000
H	2.0138711110	17.3249837570	10.0000000000
H	0.7702968499	15.1981599320	10.0000000000
C	2.4993508065	3.7265613719	10.0000000000
C	3.9223135905	3.7213245868	10.0000000000
C	4.6207482732	4.9035780180	10.0000000000
C	1.8103030997	4.9138294644	10.0000000000
H	1.9578460210	2.7812012115	10.0000000000
H	4.4566439593	2.7714159955	10.0000000000
H	5.7097503258	4.9034513903	10.0000000000
H	0.7208835784	4.9241397653	10.0000000000
P	5.0082087920	12.5156865078	10.0000000000
P	1.4703748237	12.5329159329	10.0000000000
C	4.6172828715	7.3795418192	10.0000000000
C	1.8283095832	7.3865516528	10.0000000000
H	5.7058511328	7.3770001750	10.0000000000
H	0.7391747765	7.3892417618	10.0000000000

Poly(PENT-yne)$_{2P-I}$:

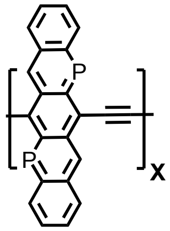

Lattice vector $|\vec{a}|$ = 6.903409099 Å

C	4.1746132260	6.2612317458	10.0000000000
C	3.9481703225	8.7494831415	10.0000000000
C	2.5069361759	8.7371419372	10.0000000000
C	2.7603105480	6.0008336149	10.0000000000
C	4.6633943011	10.0201031520	10.0000000000
C	1.7744537265	9.9804940826	10.0000000000
C	2.4891322829	11.2503431228	10.0000000000
C	3.9304906783	11.2643209572	10.0000000000
C	3.6673207532	14.0027633838	10.0000000000
C	2.2540827752	13.7366068655	10.0000000000
C	6.0518641108	10.0253370725	10.0000000000
C	7.2895887430	9.9746805437	10.0000000000
C	4.1026813182	15.3587322232	10.0000000000
C	3.1983430558	16.3971533769	10.0000000000
C	1.8032388308	16.1335404160	10.0000000000

C	1.3462433687	14.8385678531	10.0000000000
H	5.1740894223	15.5698942462	10.0000000000
H	3.5525265047	17.4280790090	10.0000000000
H	1.0962633549	16.9636262085	10.0000000000
H	0.2789997332	14.6144556686	10.0000000000
C	3.2153888339	3.6038102545	10.0000000000
C	4.6113027608	3.8610995906	10.0000000000
C	5.0760299923	5.1535791337	10.0000000000
C	2.3182040229	4.6477609878	10.0000000000
H	2.8542066791	2.5752217777	10.0000000000
H	5.3127925393	3.0269244740	10.0000000000
H	6.1452005506	5.3690084086	10.0000000000
H	1.2459000806	4.4446037579	10.0000000000
P	4.8889910625	12.7428399289	10.0000000000
P	1.5425379552	7.2634918438	10.0000000000
C	4.6906216785	7.5649449246	10.0000000000
C	1.7429324996	12.4317291864	10.0000000000
H	0.6568791100	12.3390941945	10.0000000000
H	5.7765306451	7.6549361159	10.0000000000

Poly(PENT-yne)$_{2P\text{-}II}$:

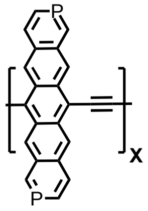

Lattice vector $|\vec{a}|$ = 6.933515850 Å

C	3.8871234390	6.2798784389	10.0000000000
C	4.5674686215	7.5056861719	10.0000000000
C	3.9190914595	8.7415698804	10.0000000000
C	2.4737485786	8.7570828884	10.0000000000
C	1.7970302870	7.5395264661	10.0000000000
C	2.4484360505	6.2920225884	10.0000000000
C	4.6728114698	9.9823146084	10.0000000000
C	1.7407343874	10.0160920669	10.0000000000
C	2.4949579225	11.2582445039	10.0000000000
C	3.9403291017	11.2400433617	10.0000000000
C	4.6224656379	12.4529770430	10.0000000000
H	5.7108921382	12.4337292489	10.0000000000
C	3.9782671960	13.7029433052	10.0000000000

C	2.5395594193	13.7218610157	10.0000000000
C	1.8523373531	12.4986009359	10.0000000000
H	5.6564735985	7.4917707200	10.0000000000
H	0.7084854665	7.5522661913	10.0000000000
H	0.7633113597	12.5191205439	10.0000000000
C	6.0515556965	9.9823194512	10.0000000000
C	7.2956796344	10.0157993752	10.0000000000
C	4.7355512340	14.9005732310	10.0000000000
C	2.4038967373	16.1982664579	10.0000000000
C	1.8265244393	14.9549981720	10.0000000000
H	5.8197000808	14.7752491945	10.0000000000
H	1.7486137263	17.0720964282	10.0000000000
H	0.7379985514	14.8817912852	10.0000000000
C	4.0455743900	3.8053848651	10.0000000000
C	4.6111966085	5.0537255188	10.0000000000
C	1.7022053990	5.0870398616	10.0000000000
H	4.7088296766	2.9376255560	10.0000000000
H	5.6988696690	5.1393768411	10.0000000000
H	0.6167972963	5.1996021845	10.0000000000
P	4.1482360290	16.5142141267	10.0000000000
P	2.3034073424	3.4778830021	10.0000000000

Poly(PENT-yne)$_{3P}$:

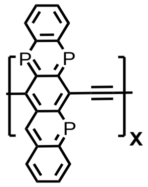

Lattice vector $|\vec{\boldsymbol{a}}|$ = 6.953074750Å

C	3.9410089923	5.9325529642	10.0000000000
C	4.1267404201	8.6726808813	10.0000000000
C	2.6786666321	8.6467757199	10.0000000000
C	2.5189814145	6.1547246088	10.0000000000
C	4.8357488999	9.9305777901	10.0000000000
C	1.9340623878	9.8901850327	10.0000000000
C	2.6520563648	11.1451931528	10.0000000000
C	4.0883492373	11.1759018198	10.0000000000
C	3.9900641288	13.9970416183	10.0000000000
C	2.5582499280	13.9480553260	10.0000000000
C	6.2421866130	9.9314028732	10.0000000000

C	7.4819604960	9.9028607675	10.0000000000
C	4.6114368141	15.2832232677	10.0000000000
C	3.8740300698	16.4444057954	10.0000000000
C	2.4574060582	16.3912577667	10.0000000000
C	1.8241351769	15.1731897280	10.0000000000
H	5.6999533443	15.3389511390	10.0000000000
H	4.3801788302	17.4094421292	10.0000000000
H	1.8744071442	17.3126135672	10.0000000000
H	0.7348037369	15.1210521464	10.0000000000
C	2.1464215798	3.7429660179	10.0000000000
C	3.5503941618	3.5241268769	10.0000000000
C	4.4194622319	4.5903769089	10.0000000000
C	1.6488154018	5.0222328219	10.0000000000
H	1.4679501532	2.8898198103	10.0000000000
H	3.9388059332	2.5055106101	10.0000000000
H	5.4970829869	4.4137063382	10.0000000000
H	0.5757615413	5.2144852350	10.0000000000
P	5.0953276182	12.6281748406	10.0000000000
P	1.5514636717	12.5196415535	10.0000000000
C	1.9665977360	7.4409135132	10.0000000000
H	0.8779088117	7.5031337576	10.0000000000
P	5.1276787921	7.2199453918	10.0000000000

Poly(PENT-yne)$_{4P}$:

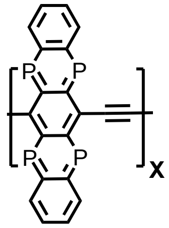

Lattice vector $|\vec{a}|$ = 7.003228836 Å

C	3.9516896576	5.9406552730	10.0000000000
C	3.9558344170	8.7538019295	10.0000000000
C	2.5124398041	8.7571841564	10.0000000000
C	2.5169037289	5.9414550554	10.0000000000
C	4.6934009397	9.9953435857	10.0000000000
C	1.7791994530	10.0030156324	10.0000000000
C	2.5173829215	11.2457294115	10.0000000000

C	3.9607848112	11.2400281426	10.0000000000
C	3.9778300867	14.0517304887	10.0000000000
C	2.5430256312	14.0628798001	10.0000000000
C	6.1173051139	9.9960352622	10.0000000000
C	7.3588450256	10.0022231039	10.0000000000
C	4.6685389272	15.3028280899	10.0000000000
C	3.9941929372	16.4997255467	10.0000000000
C	2.5766686639	16.5119892094	10.0000000000
C	1.8781036388	15.3282430188	10.0000000000
H	5.7591493292	15.2940705434	10.0000000000
H	4.5443600095	17.4406690990	10.0000000000
H	2.0447322476	17.4633720552	10.0000000000
H	0.7877551379	15.3453429303	10.0000000000
C	2.5268058962	3.4924270149	10.0000000000
C	3.9443254796	3.4922175851	10.0000000000
C	4.6302746211	4.6827542603	10.0000000000
C	1.8397054180	4.6827724418	10.0000000000
H	1.9858395021	2.5461720427	10.0000000000
H	4.4856680685	2.5461031533	10.0000000000
H	5.7208219737	4.6798667155	10.0000000000
H	0.7492805710	4.6776783946	10.0000000000
P	5.0262368519	12.6473851041	10.0000000000
P	1.4728611203	12.6715043824	10.0000000000
P	5.0108414309	7.3378318938	10.0000000000
P	1.4573601852	7.3400242176	10.0000000000

Poly(PENT-yne)$_{5P}$:

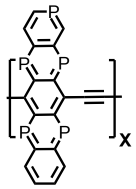

Lattice vector $|\vec{\boldsymbol{a}}|$ = 7.00322884 Å

C	3.9621018265	5.9344237134	10.0000000000
C	3.9783176230	8.7498336666	10.0000000000
C	2.5359202421	8.7572513182	10.0000000000
C	2.5286041875	5.9447876075	10.0000000000
C	4.7153055148	9.9932147855	10.0000000000

C	1.8027638941	10.0050233278	10.0000000000
C	2.5401223082	11.2460235896	10.0000000000
C	3.9819786192	11.2387409629	10.0000000000
C	3.9917406473	14.0689066364	10.0000000000
C	2.5576736227	14.0746262201	10.0000000000
C	6.1369185407	9.9956825504	10.0000000000
C	7.3781132093	10.0031813484	10.0000000000
C	4.7303021230	15.2865938707	10.0000000000
C	2.3972029851	16.5664379454	10.0000000000
C	1.8396215148	15.3145846059	10.0000000000
H	5.8189606129	15.1865516433	10.0000000000
H	1.7267103921	17.4288023712	10.0000000000
H	0.7491095134	15.2414913497	10.0000000000
C	3.9353323331	3.4847214441	10.0000000000
C	4.6298457183	4.6712600085	10.0000000000
C	1.8415435494	4.6930587698	10.0000000000
H	4.4709842010	2.5349500000	10.0000000000
H	5.7200560233	4.6603005362	10.0000000000
H	0.7513684897	4.7006525058	10.0000000000
P	5.0369316158	12.6528007812	10.0000000000
P	1.4987030097	12.6756466889	10.0000000000
P	5.0273779502	7.3284621988	10.0000000000
P	1.4760051390	7.3474996955	10.0000000000
P	4.1373697638	16.8980061831	10.0000000000
C	2.5177879600	3.4963165139	10.0000000000
H	1.9662343365	2.5565127413	10.0000000000

Poly(PENT-yne)$_{6P}$:

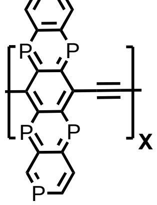

Lattice vector $|\vec{a}|$ = 6.996866890 Å

C	3.9193159609	5.9216304964	10.0000000000
C	3.9501018131	8.7463349535	10.0000000000
C	2.5083793909	8.7631426791	10.0000000000
C	2.4857252445	5.9342723815	10.0000000000

C	4.6926877627	9.9837497094	10.0000000000
C	1.7805144923	10.0147287888	10.0000000000
C	2.5235312187	11.2530433121	10.0000000000
C	3.9652919803	11.2345404188	10.0000000000
C	4.0030538602	14.0605203869	10.0000000000
C	2.5696183465	14.0806640384	10.0000000000
C	6.1145734712	9.9871261401	10.0000000000
C	7.3557493069	10.0108308128	10.0000000000
C	4.7583874577	15.2673550781	10.0000000000
C	2.4437689330	16.5730178785	10.0000000000
C	1.8679297832	15.3293150394	10.0000000000
H	5.8452897538	15.1501168086	10.0000000000
H	1.7828229509	17.4422739288	10.0000000000
H	0.7765028232	15.2752220190	10.0000000000
C	4.0658919531	3.4306776337	10.0000000000
C	4.6309704601	4.6788833734	10.0000000000
C	1.7406350555	4.7210884199	10.0000000000
H	4.7340902336	2.5669335197	10.0000000000
H	5.7219291368	4.7438917589	10.0000000000
H	0.6526416839	4.8271108183	10.0000000000
P	5.0340207051	12.6357670132	10.0000000000
P	1.4948027879	12.6928171720	10.0000000000
P	4.9863388500	7.3135868892	10.0000000000
P	1.4467829335	7.3550356839	10.0000000000
P	4.1869001516	16.8859835520	10.0000000000
P	2.3248228093	3.1065576860	10.0000000000

II:

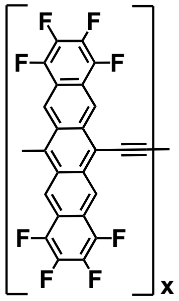

Lattice vector $|\vec{\boldsymbol{a}}|$ = 6.935162327Å

C	3.9791724438	8.7988453234	12.5000000000
C	4.6518996046	10.0256007483	12.5000000000
C	3.9841345185	11.2505565903	12.5000000000

C	2.5383951961	11.2534219466	12.5000000000
C	1.8680082426	10.0285244710	12.5000000000
C	2.5395824563	8.7992843770	12.5000000000
C	4.7288907781	12.4955341967	12.5000000000
C	1.7991176904	12.5041978221	12.5000000000
C	2.5442374483	13.7512210990	12.5000000000
C	3.9899666048	13.7442380934	12.5000000000
C	4.6661721054	14.9640786143	12.5000000000
H	5.7539613730	14.9515320032	12.5000000000
C	4.0025758989	16.1952382118	12.5000000000
C	2.5630368085	16.2059699895	12.5000000000
C	1.8823182712	14.9812283526	12.5000000000
H	5.7398497083	10.0302168042	12.5000000000
H	0.7795885442	10.0353130998	12.5000000000
H	0.7937673267	14.9826630994	12.5000000000
C	6.1098377822	12.4953950070	12.5000000000
C	7.3532081070	12.5043906702	12.5000000000
C	4.7079283598	17.4274911599	12.5000000000
C	4.0236764486	18.6104969074	12.5000000000
C	2.6020034086	18.6231084975	12.5000000000
C	1.8883326575	17.4564055961	12.5000000000
C	2.5560300998	6.3823326623	12.5000000000
C	3.9777273342	6.3829930516	12.5000000000
C	4.6728898592	7.5597591930	12.5000000000
C	1.8532803297	7.5554478251	12.5000000000
F	4.6766229559	19.7916169898	12.5000000000
F	1.9759925285	19.8189862210	12.5000000000
F	4.6200439250	5.1960470091	12.5000000000
F	1.9192169756	5.1921973457	12.5000000000
H	5.7942445802	17.4356615129	12.5000000000
H	0.8028881831	17.4994864423	12.5000000000
H	5.7590411949	7.5395298113	12.5000000000
H	0.7674683339	7.5240973856	12.5000000000

III:

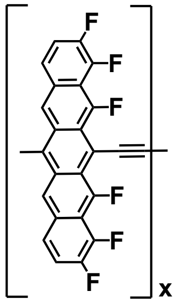

Lattice vector $|\vec{a}|$ = 7.073216230 Å

C	4.0915648529	8.7422472268	12.5000000000
C	4.7922987720	9.9682247953	12.5000000000
C	4.2213351396	11.2515093314	12.5000000000
C	2.7602996266	11.2673592683	12.5000000000
C	2.0571398962	10.0724529400	12.5000000000
C	2.6608282482	8.8151458172	12.5000000000
C	5.0145654647	12.4958703690	12.5000000000
C	1.9939498181	12.5023279285	12.5000000000
C	2.7637484053	13.7350497313	12.5000000000
C	4.2249874767	13.7425765320	12.5000000000
C	4.8027624206	15.0223896857	12.5000000000
C	4.1094882896	16.2518693329	12.5000000000
C	2.6786001097	16.1887353566	12.5000000000
C	2.0671051422	14.9343626882	12.5000000000
H	0.9772176393	10.1170582639	12.5000000000
H	0.9870722509	14.8956294412	12.5000000000
C	6.4167223683	12.4959347643	12.5000000000
C	7.6704438669	12.5013015846	12.5000000000
C	4.6777050708	17.5527513033	12.5000000000
C	3.8891521349	18.6847325783	12.5000000000
C	2.4865170383	18.6128783654	12.5000000000
C	1.9006544804	17.3719645406	12.5000000000
C	2.4476471819	6.3936954675	12.5000000000
C	3.8500890085	6.3110789130	12.5000000000
C	4.6488508837	7.4362416689	12.5000000000
C	1.8723605132	7.6392739271	12.5000000000
F	6.0175880961	17.7433362981	12.5000000000
F	4.4922895810	19.8971072840	12.5000000000
F	5.9872984257	7.2338536501	12.5000000000
F	4.4422212482	5.0932796252	12.5000000000
H	0.8256606196	17.2614454634	12.5000000000
H	1.9056176165	19.5338257290	12.5000000000
H	0.7980797210	7.7606118545	12.5000000000
H	1.8589746942	5.4778675370	12.5000000000
F	6.1542614441	15.1235475931	12.5000000000
F	6.1437770818	9.8600948142	12.5000000000

IV

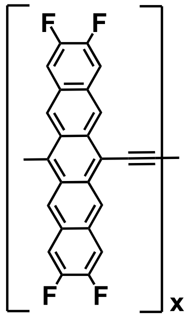

Lattice vector $|\vec{a}|$ = 6.935162327 Å

C	3.9791724438	8.7988453234	12.5000000000
C	4.6518996046	10.0256007483	12.5000000000
C	3.9841345185	11.2505565903	12.5000000000
C	2.5383951961	11.2534219466	12.5000000000
C	1.8680082426	10.0285244710	12.5000000000
C	2.5395824563	8.7992843770	12.5000000000
C	4.7288907781	12.4955341967	12.5000000000
C	1.7991176904	12.5041978221	12.5000000000
C	2.5442374483	13.7512210990	12.5000000000
C	3.9899666048	13.7442380934	12.5000000000
C	4.6661721054	14.9640786143	12.5000000000
H	5.7539613730	14.9515320032	12.5000000000
C	4.0025758989	16.1952382118	12.5000000000
C	2.5630368085	16.2059699895	12.5000000000
C	1.8823182712	14.9812283526	12.5000000000
H	5.7398497083	10.0302168042	12.5000000000
H	0.7795885442	10.0353130998	12.5000000000
H	0.7937673267	14.9826630994	12.5000000000
C	6.1098377822	12.4953950070	12.5000000000
C	7.3532081070	12.5043906702	12.5000000000
C	4.7079283598	17.4274911599	12.5000000000
C	4.0236764486	18.6104969074	12.5000000000
C	2.6020034086	18.6231084975	12.5000000000
C	1.8883326575	17.4564055961	12.5000000000
C	2.5560300998	6.3823326623	12.5000000000
C	3.9777273342	6.3829930516	12.5000000000
C	4.6728898592	7.5597591930	12.5000000000
C	1.8532803297	7.5554478251	12.5000000000
F	4.6766229559	19.7916169898	12.5000000000
F	1.9759925285	19.8189862210	12.5000000000
F	4.6200439250	5.1960470091	12.5000000000
F	1.9192169756	5.1921973457	12.5000000000
H	5.7942445802	17.4356615129	12.5000000000

H	0.8028881831	17.4994864423	12.5000000000
H	5.7590411949	7.5395298113	12.5000000000
H	0.7674683339	7.5240973856	12.5000000000

V:

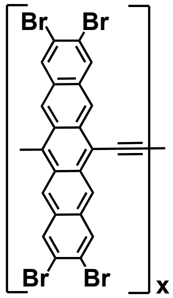

Lattice vector $|\vec{a}|$ = 6.976068486Å

C	4.0351744511	8.7987161819	12.5000000000
C	4.7112670083	10.0252072438	12.5000000000
C	4.0453994815	11.2518799109	12.5000000000
C	2.5928945468	11.2551262568	12.5000000000
C	1.9242426146	10.0287076796	12.5000000000
C	2.5984138645	8.7995236185	12.5000000000
C	4.7985549025	12.4954600293	12.5000000000
C	1.8454344124	12.5046578351	12.5000000000
C	2.5988871617	13.7503142790	12.5000000000
C	4.0514132770	13.7429119034	12.5000000000
C	4.7261777572	14.9640812146	12.5000000000
H	5.8151038346	14.9557357275	12.5000000000
C	4.0596774977	16.1951136111	12.5000000000
C	2.6229651076	16.2065389557	12.5000000000
C	1.9392287262	14.9820888848	12.5000000000
H	5.8002829352	10.0253766212	12.5000000000
H	0.8351796761	10.0309172902	12.5000000000
H	0.8500857310	14.9885773283	12.5000000000
C	6.1865848126	12.4955012637	12.5000000000
C	7.4336719775	12.5046378057	12.5000000000
C	4.7569095331	17.4296686698	12.5000000000
C	4.0816320241	18.6267187397	12.5000000000
C	2.6553755140	18.6408402441	12.5000000000
C	1.9528860129	17.4579056583	12.5000000000
C	2.6068741898	6.3654659500	12.5000000000
C	4.0331451465	6.3661754831	12.5000000000
C	4.7204242600	7.5568473276	12.5000000000
C	1.9162432024	7.5550669720	12.5000000000

H	5.8438483934	17.4225412825	12.5000000000
H	0.8660291404	17.4786792583	12.5000000000
H	5.8073102914	7.5520904079	12.5000000000
H	0.8292759511	7.5457058670	12.5000000000
Br	5.0677928877	20.2430177152	12.5000000000
Br	1.7162900912	20.2857594102	12.5000000000
Br	5.0000488315	4.7379677452	12.5000000000
Br	1.6486417840	4.7318497181	12.5000000000

VI:

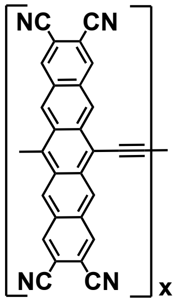

Lattice vector $|\vec{\boldsymbol{a}}|$ = 6.951305330 Å

C	4.0243027210	8.7989812872	12.5000000000
C	4.6987830126	10.0253116885	12.5000000000
C	4.0319944833	11.2511897140	12.5000000000
C	2.5824448781	11.2543863857	12.5000000000
C	1.9131576816	10.0285605946	12.5000000000
C	2.5858627751	8.7997509870	12.5000000000
C	4.7795763494	12.4953868325	12.5000000000
C	1.8410561943	12.5047231434	12.5000000000
C	2.5889740872	13.7509293381	12.5000000000
C	4.0385396406	13.7437154069	12.5000000000
C	4.7145720052	14.9640877326	12.5000000000
H	5.8029824649	14.9564966943	12.5000000000
C	4.0499329547	16.1952065994	12.5000000000
C	2.6115243420	16.2060413995	12.5000000000
C	1.9289866637	14.9821766555	12.5000000000
H	5.7872722992	10.0241634160	12.5000000000
H	0.8245860438	10.0289236275	12.5000000000
H	0.8403529376	14.9909798185	12.5000000000
C	6.1638479822	12.4952035068	12.5000000000
C	7.4081494538	12.5049321568	12.5000000000
C	4.7482055728	17.4274273831	12.5000000000
C	4.0784774110	18.6331686897	12.5000000000
C	2.6347016062	18.6464162579	12.5000000000
C	1.9394894186	17.4539898105	12.5000000000

C	2.5864530669	6.3595742812	12.5000000000
C	4.0302526836	6.3603153027	12.5000000000
C	4.7110362883	7.5600798456	12.5000000000
C	1.9022619729	7.5582132596	12.5000000000
H	5.8350507544	17.4206327219	12.5000000000
H	0.8528675261	17.4748298576	12.5000000000
H	5.7977920311	7.5553374138	12.5000000000
H	0.8154766117	7.5485518493	12.5000000000
C	4.7804743398	19.8714222147	12.5000000000
N	5.3151386262	20.9080889222	12.5000000000
C	1.9626435211	19.9010839084	12.5000000000
N	1.4622510493	20.9549425884	12.5000000000
C	4.7207847088	5.1157721972	12.5000000000
N	5.2451778874	4.0736328922	12.5000000000
C	1.9028048227	5.1111466168	12.5000000000
N	1.3920374974	4.0623160623	12.5000000000

VII:

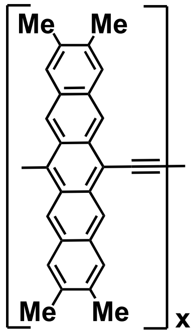

Lattice vector $|\vec{\boldsymbol{a}}|$ = 6.941824064 Å

C	4.0170340446	8.8002343687	12.5000119669
C	4.6905728759	10.0261417684	12.5000064416
C	4.0231322741	11.2513424307	12.5000012877
C	2.5752227266	11.2542693362	12.4999990019
C	1.9061106606	10.0279800570	12.4999987208
C	2.5798547113	8.7999123433	12.5000087395
C	4.7694789295	12.4948316885	12.4999993765
C	1.8364225935	12.5050943983	12.4999997450
C	2.5832568582	13.7510824691	12.5000001868
C	4.0311228077	13.7432404397	12.4999996094
C	4.7087021062	14.9627378877	12.4999994052
H	5.7975544200	14.9532167871	12.4999999619
C	4.0457587911	16.1940712617	12.4999988886
C	2.6086755492	16.2058346156	12.4999991447
C	1.9245466677	14.9830924715	12.5000002097

H	5.7795489224	10.0257276723	12.4999999978
H	0.8168907832	10.0280214308	12.4999984006
H	0.8354019636	14.9922951559	12.5000001507
C	6.1520539014	12.4941528098	12.4999998750
C	7.3957921761	12.5052901489	12.5000001061
C	4.7369003234	17.4339969162	12.5000026885
C	4.0740213248	18.6400389275	12.5000039671
C	2.6384982734	18.6540793740	12.4999986943
C	1.9473516594	17.4627296613	12.4999985388
C	2.5887171568	6.3517099807	12.5000128360
C	4.0243659299	6.3541081240	12.4999600286
C	4.6974785606	7.5545074987	12.4999732437
C	1.9075555133	7.5487651351	12.5000144506
H	5.8269211572	17.4190679796	12.5000038746
H	0.8575132891	17.4780446943	12.4999993150
H	5.7875813239	7.5601066025	12.4999610045
H	0.8175021225	7.5430350388	12.5000063374
C	4.8270523508	19.9382829562	12.5000026529
H	4.5809370914	20.5493154258	11.6176906477
H	4.5806468951	20.5494874220	13.3821115396
H	5.9062845342	19.7568074724	12.5001979406
C	1.9157012531	19.9691686233	12.4999980062
H	2.1772586617	20.5737847140	13.3823271868
H	2.1773074620	20.5738120668	11.6177026965
H	0.8325665583	19.8138607966	12.4999644637
C	4.7669548476	5.0499224534	12.4999733921
H	4.5174254721	4.4417861703	13.3833113071
H	4.5145067639	4.4400861375	11.6186689791
H	5.8475754143	5.2227498190	12.4980187281
C	1.8550557621	5.0425898785	12.5000197607
H	2.1112360871	4.4358482035	11.6175211164
H	2.1117111454	4.4355764793	13.3821870046
H	0.7732986386	5.2074308676	12.5003483922

VII:

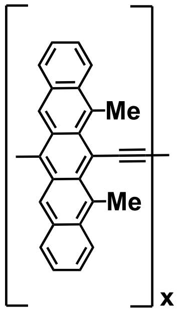

Lattice vector $|\vec{a}|$ = 7.205618326 Å

C	4.0255028323	8.7211014083	12.5000075638
C	4.7752592356	9.9368357380	12.5000112757
C	4.2080132849	11.2391093888	12.5000247039
C	2.7411522687	11.2754054641	12.5000300417
C	2.0395824677	10.0789242241	12.5000173477
C	2.6039431339	8.8019678053	12.5000082347
C	5.0261947525	12.4950558661	12.5000279205
C	1.9441331714	12.5003598234	12.5000440698
C	2.7450877618	13.7236450243	12.5000332189
C	4.2129369046	13.7541798117	12.5000136820
C	4.7863017525	15.0540949964	12.4999709679
C	4.0425935056	16.2732213108	12.5000046852
C	2.6206291513	16.1986381511	12.5000180608
C	2.0504249553	14.9240314656	12.5000213325
C	6.4604415806	12.4935027003	12.5000498661
C	7.7301559070	12.4975839195	12.5000567075
C	4.6245274499	17.5800081177	12.5000242351
C	3.8501506742	18.7185311886	12.5000211471
C	2.4387942990	18.6281629042	12.5000072628
C	1.8433531161	17.3902436857	12.5000162447
C	2.4098827380	6.3732099766	12.4999979197
C	3.8204948175	6.2763109158	12.4999984460
C	4.6006196532	7.4111324460	12.5000016672
C	1.8209413363	7.6141938888	12.5000012637
H	5.7018929568	17.7213612339	12.5000549141
H	0.7599203041	17.3133832179	12.5000255012
H	5.6771135493	7.2641817492	12.5000005555
H	0.7384608531	7.6958300609	12.4999994766
C	6.2477197160	15.2030868141	12.4998874238
H	6.6765538176	14.7243937211	13.3880439564
H	6.6764949857	14.7241848627	11.6118183595
H	6.5584108842	16.2412834672	12.4997349030
C	6.2359419638	9.7831185078	12.4999975063
H	6.6665654249	10.2596886116	11.6113813574
H	6.6665746391	10.2596493594	13.3886304209
H	6.5415905862	8.7436976635	12.4999712423
H	4.3356487309	19.6952388059	12.5000328479
H	1.8321028055	19.5339714350	12.4999960992
H	4.3005647616	5.2969194597	12.4999958755
H	1.7977742094	5.4710377209	12.4999948104
H	0.9834055946	14.8512538016	12.5000120336

H	0.9729440086	10.1588663463	12.5000148512

Heptacene Polymer:

Poly(HEPT-yne)(n=7):

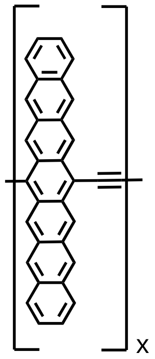

Lattice vector $|\vec{a}|$ = 6.954678983Å

C	3.9881961552	11.2928735164	15.0000000000
C	4.6464427017	12.5371882242	15.0000000000
C	3.9750176434	13.7496034169	15.0000000000
C	2.5187500257	13.7437105665	15.0000000000
C	1.8626505072	12.5172992288	15.0000000000
C	2.5422722842	11.2773957498	15.0000000000
C	4.7224388734	14.9988279052	15.0000000000
C	1.7732975242	14.9995801659	15.0000000000
C	2.5226337081	16.2539180714	15.0000000000
C	3.9789587128	16.2499047971	15.0000000000
C	4.6524068506	17.4624713757	15.0000000000
H	5.7412285962	17.4499390508	15.0000000000
C	3.9946474805	18.7081392975	15.0000000000
C	2.5484383999	18.7192723107	15.0000000000
C	1.8685759917	17.4799984413	15.0000000000
H	5.7349416965	12.5456072605	15.0000000000
H	0.7736132247	12.5107735234	15.0000000000
H	0.7797002012	17.4885384169	15.0000000000
C	6.1018998750	14.9978838571	15.0000000000
C	7.3488498975	15.0001533370	15.0000000000
C	4.6912859090	19.9245696199	15.0000000000
C	4.0267966072	21.1533045336	15.0000000000
C	2.5788986911	21.1699765136	15.0000000000
C	1.8833882324	19.9548539666	15.0000000000

H	5.7807251702	19.9103213939	15.0000000000
H	0.7940426428	19.9665925070	15.0000000000
C	2.5905660317	8.8263231848	15.0000000000
C	4.0381600276	8.8495923075	15.0000000000
C	4.6933735755	10.0821916428	15.0000000000
C	1.8854541586	10.0366035789	15.0000000000
H	5.7823651655	10.1094144666	15.0000000000
H	0.7962659173	10.0113684573	15.0000000000
C	4.7266167885	22.3947661321	15.0000000000
C	4.0533791972	23.5898235648	15.0000000000
C	2.6299292494	23.6091484902	15.0000000000
C	1.9194108848	22.4346207244	15.0000000000
H	5.8154649457	22.3711763694	15.0000000000
H	4.6057176404	24.5291451202	15.0000000000
H	2.1067604126	24.5655248704	15.0000000000
H	0.8309099557	22.4532277991	15.0000000000
C	2.6545150969	6.3873919534	15.0000000000
C	4.0781022688	6.4138762008	15.0000000000
C	4.7453278825	7.6123361992	15.0000000000
C	1.9382515110	7.5582908302	15.0000000000
H	2.1361464211	5.4285013441	15.0000000000
H	4.6352235589	5.4774300824	15.0000000000
H	5.8340625622	7.6421590583	15.0000000000
H	0.8497936671	7.5357066952	15.0000000000

Nonacene Polymer:

Poly(NONA-yne) (n=9):

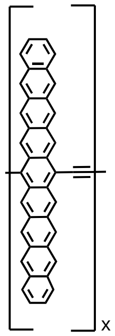

Lattice vector $|\vec{a}|$ = 6.974296293Å

C	4.0568493571	13.7894425453	17.5000000000
C	4.7153858242	15.0390432675	17.5000000000

C	4.0462672524	16.2510977302	17.5000000000
C	2.5840843232	16.2461113156	17.5000000000
C	1.9298569834	15.0234134237	17.5000000000
C	2.6062026648	13.7793977240	17.5000000000
C	4.7938577729	17.5017333722	17.5000000000
C	1.8331903979	17.4984852190	17.5000000000
C	2.5821286910	18.7521981410	17.5000000000
C	4.0442972955	18.7509678451	17.5000000000
C	4.7109670481	19.9647024942	17.5000000000
H	5.8000972368	19.9577466278	17.5000000000
C	4.0501443898	21.2130588648	17.5000000000
C	2.5995239311	21.2183270203	17.5000000000
C	1.9253741428	19.9731745198	17.5000000000
H	5.8046589840	15.0491027390	17.5000000000
H	0.8404907614	15.0178954005	17.5000000000
H	0.8358671625	19.9757873612	17.5000000000
C	6.1767489460	17.5018063789	17.5000000000
C	7.4245304200	17.4985597252	17.5000000000
C	4.7403577736	22.4249779950	17.5000000000
C	4.0695606799	23.6625506354	17.5000000000
C	2.6148798457	23.6704257372	17.5000000000
C	1.9279212950	22.4407916848	17.5000000000
H	5.8302430575	22.4117796473	17.5000000000
H	0.8389124926	22.4482507126	17.5000000000
C	2.6292067761	11.3266362678	17.5000000000
C	4.0839025189	11.3404291467	17.5000000000
C	4.7503817060	12.5799596117	17.5000000000
C	1.9380678865	12.5544238466	17.5000000000
H	5.8399711362	12.5964952313	17.5000000000
H	0.8487727712	12.5436406472	17.5000000000
C	4.7530593816	24.8911726783	17.5000000000
C	4.0795277508	26.1134851303	17.5000000000
C	2.6296961268	26.1218083199	17.5000000000
C	1.9444679571	24.9062941154	17.5000000000
H	5.8427695175	24.8874145949	17.5000000000
H	0.8549398010	24.9115502196	17.5000000000
C	2.6571288660	8.8744283720	17.5000000000
C	4.1069433105	8.8902155461	17.5000000000

C	4.7736511298	10.1156596337	17.5000000000
C	1.9652513602	10.0868678519	17.5000000000
H	5.8632642948	10.1251561617	17.5000000000
H	0.8756448086	10.0757410852	17.5000000000
C	4.7654761961	27.3648151770	17.5000000000
C	4.0793609483	28.5521553852	17.5000000000
C	2.6537143391	28.5601357473	17.5000000000
C	1.9556498704	27.3801866737	17.5000000000
H	5.8543133308	27.3542017060	17.5000000000
H	4.6219376433	29.4975325722	17.5000000000
H	2.1220404888	29.5119640901	17.5000000000
H	0.8662489175	27.3847367294	17.5000000000
C	2.6986648330	6.4357934431	17.5000000000
C	4.1242616574	6.4520588264	17.5000000000
C	4.8018325524	7.6438772153	17.5000000000
C	1.9921672956	7.6110684867	17.5000000000
H	2.1732475153	5.4805720124	17.5000000000
H	4.6728818970	5.5100676817	17.5000000000
H	5.8907597468	7.6630797605	17.5000000000
H	0.9029623108	7.5976820551	17.5000000000

Bisanthene Polymer:

Poly(BISANTH-yne):

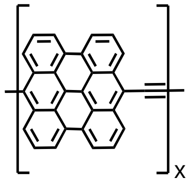

Lattice vector $|\vec{a}|$ = 11.200596444 Å

C	8.1127213819	3.8267663438	7.5000000000
C	8.8029714718	5.0273313008	7.5000000000
C	8.1214697855	6.2556643459	7.5000000000
C	6.6889997770	6.2617298630	7.5000000000
C	5.9857966853	5.0178600640	7.5000000000
C	6.7208898838	3.8220256779	7.5000000000
C	8.8565797543	7.5028100049	7.5000000000
C	5.9652733026	7.5013976215	7.5000000000
C	6.6873923273	8.7420755582	7.5000000000
C	8.1199724670	8.7492882758	7.5000000000

C	8.7998889774	9.9783714135	7.5000000000
H	9.8870137398	9.9708785935	7.5000000000
C	8.1081453479	11.1782222080	7.5000000000
C	6.7162968111	11.1822006637	7.5000000000
C	5.9827544060	9.9853306795	7.5000000000
H	8.6588289715	2.8834485251	7.5000000000
H	9.8900927478	5.0359382601	7.5000000000
H	6.2112997202	2.8620102553	7.5000000000
H	8.6534225165	12.1220141608	7.5000000000
H	6.2049934963	12.1416484229	7.5000000000
C	10.2363290762	7.5022470965	7.5000000000
C	11.4767907617	7.4975040408	7.5000000000
C	3.7933520508	3.8199032298	7.5000000000
C	4.5270860301	5.0168616870	7.5000000000
C	3.8236817175	6.2598675870	7.5000000000
C	2.3916120295	6.2517863737	7.5000000000
C	1.7112407758	5.0227843054	7.5000000000
C	2.4015113936	3.8221623380	7.5000000000
C	4.5462052034	7.5003413930	7.5000000000
C	1.6556940757	7.4976805569	7.5000000000
C	2.3900931209	8.7449631187	7.5000000000
C	3.8221985950	8.7397612381	7.5000000000
C	4.5234391889	9.9841258198	7.5000000000
C	3.7867688744	11.1793013976	7.5000000000
C	2.3949504123	11.1739103936	7.5000000000
C	1.7071080565	9.9720104100	7.5000000000
H	4.3056352373	2.8616578578	7.5000000000
H	0.6245773320	5.0299472471	7.5000000000
H	1.8576145938	2.8773434563	7.5000000000
H	4.2971946871	12.1386894039	7.5000000000
H	1.8481995927	12.1171481464	7.5000000000
H	0.6204520063	9.9617054944	7.5000000000

Peripentacene Polymer:

Poly(PERIPENT-yne):

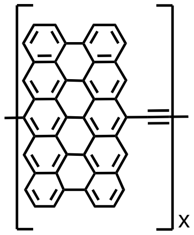

Lattice vector $|\vec{\boldsymbol{a}}|$ = 11.218757645 Å

C	8.2226990940	8.7971134025	12.4536404147
C	8.9061252302	10.0283139910	12.4961378244
C	8.2491173948	11.2454387901	12.5276202019
C	6.8065796721	11.2617701834	12.5281332059
C	6.0943513911	10.0374932407	12.5102422362
C	6.7919316397	8.7900141364	12.4726989733
C	8.9948523008	12.4952219366	12.5419934094
C	6.0917242792	12.5011365687	12.5369526022
C	6.8109705167	13.7378159399	12.5369502641
C	8.2532022685	13.7477714323	12.5369507013
C	8.9148031852	14.9626108984	12.5200007231
H	10.0034253711	14.9679515560	12.5141140596
C	8.2366721025	16.1973542684	12.4991370312
C	6.8061835527	16.2104693004	12.5108448650
C	6.1037614468	14.9652728438	12.5292591846
H	9.9949631043	10.0188484845	12.4890118592
C	10.3678545193	12.4960074210	12.5480143113
C	11.6126667994	12.5078898596	12.5479735910
C	3.9546587392	8.7957724744	12.4985340599
C	4.6579907998	10.0403057197	12.5128722781
C	3.9506343037	11.2676419498	12.5280037034
C	2.5086900060	11.2570235090	12.5332112864
C	1.8464884328	10.0426767987	12.5303064707
C	2.5240285922	8.8080095727	12.5152520085
C	4.6699900070	12.5041292802	12.5352645776
C	1.7669877564	12.5091524402	12.5426733703
C	2.5132630695	13.7589177335	12.5389187140
C	3.9555532440	13.7434611896	12.5360209919
C	4.6677279389	14.9684010034	12.5303214798
C	3.9703177138	16.2165149746	12.5258159778

C	2.5395290352	16.2086630646	12.5355900940
C	1.8565779612	14.9762016434	12.5390028867
H	0.7581003365	10.0390705693	12.5378641931
H	0.7678675019	14.9850440594	12.5419255273
C	2.4891361838	6.3843730729	12.4972106532
C	3.8879702102	6.3655528486	12.4779442777
C	4.6407505345	7.5460945045	12.4708708546
C	1.8124737988	7.5888617663	12.5123749981
H	1.9379681201	5.4444371811	12.4991006352
H	4.3895388226	5.4005073810	12.4733525290
H	0.7250123533	7.6179941474	12.5289114715
C	6.8470274389	6.3616026828	12.3556767118
C	8.2457191584	6.3765042717	12.3230616782
C	8.9276535391	7.5764433674	12.3790142189
C	6.1002530653	7.5437351713	12.4360230682
H	6.3407003856	5.4002280376	12.3090981669
H	8.7924691464	5.4364921771	12.2507277978
H	10.0151199136	7.6028983870	12.3505108786
C	4.6613556035	17.4639701853	12.5152228729
C	3.9140173006	18.6483823508	12.5281969512
C	2.5151521907	18.6332070176	12.5414446653
C	1.8341719667	17.4313088846	12.5418617577
H	4.4184804566	19.6121310913	12.5328024534
H	1.9674040740	19.5752331739	12.5522561013
H	0.7464175312	17.4071689971	12.5535258633
C	8.9481109723	17.4158682659	12.4608268592
C	8.2721277149	18.6207881681	12.4337024916
C	6.8733559573	18.6410807394	12.4514251682
C	6.1207841665	17.4608993654	12.4936812674
H	10.0355727652	17.3847807872	12.4448899434
H	8.8238522609	19.5597262199	12.3982663258
H	6.3719868385	19.6062051906	12.4281968417

References:

(1) Ji, L.; Friedrich, A.; Krummenacher, I.; Eichhorn, A.; Braunschweig, H.; Moos, M.; Hahn, S.; Geyer, F. L.; Tverskoy, O.; Han, J.; et al. Preparation, Properties, and Structures of the Radical Anions and Dianions of Azapentacenes. *J. Am. Chem. Soc.* **2017**, *139* (44), 15968-15976. DOI: 10.1021/jacs.7b09460.

(2) Liang, Z.; Tang, Q.; Xu, J.; Miao, Q. Soluble and stable N-heteropentacenes with high field-effect mobility. *Adv. Mater.* **2011**, *23* (13), 1535-1539.
(3) Liu, H.; Neal, A. T.; Zhu, Z.; Luo, Z.; Xu, X.; Tománek, D.; Ye, P. D. Phosphorene: an unexplored 2D semiconductor with a high hole mobility. *ACS nano* **2014**, *8* (4), 4033-4041.
(4) Long, G.; Yang, X.; Chen, W.; Zhang, M.; Zhao, Y.; Chen, Y.; Zhang, Q. "Doping" pentacene with sp^2-phosphorus atoms: towards high performance ambipolar semiconductors. *Phys. Chem. Chem. Phys.* **2016**, *18* (4), 3173-3178.
(5) Hong, S. Y.; Kertesz, M. Dependence of Young's modulus of trans-polyacetylene upon charge transfer. *Phys. Rev. Lett.* **1990**, *64* (25), 3031.
(6) Kurita, T.; Fukuda, Y.; Takahashi, M.; Sasanuma, Y. Crystalline moduli of polymers, evaluated from density functional theory calculations under periodic boundary conditions. *ACS omega* **2018**, *3* (5), 4824-4835.
(7) Li, P.; Hu, L.; McGaughey, A. J.; Shen, S. Crystalline polyethylene nanofibers with the theoretical limit of Young's modulus. *Adv. Mater.* **2014**, *26* (7), 1065-1070.
(8) Chudinov, V. S.; Shardakov, I. N.; Ivanov, Y. N.; Morozov, I. A.; Belyaev, A. Y. Elastic Modulus of a Carbonized Layer on Polyurethane Treated by Ion-Plasma. *Polymers* **2023**, *15* (6), 1442.
(9) Akagi, K.; Sakamaki, K.; Shirakawa, H.; Kyotani, H. Polyacetylene films prepared by intrinsic non-solvent polymerization method-mechanical properties and electrical conductivities. *Synth. Met.* **1995**, *69* (1-3), 29-30.
